# A distinct tumor microenvironment makes anaplastic thyroid cancer more lethal but immunotherapy sensitive than papillary thyroid cancer

**DOI:** 10.1172/jci.insight.173712

**Published:** 2024-03-13

**Authors:** Pei-Zhen Han, Wei-Dong Ye, Peng-Cheng Yu, Li-Cheng Tan, Xiao Shi, Xu-Feng Chen, Cong He, Jia-Qian Hu, Wen-Jun Wei, Zhong-Wu Lu, Ning Qu, Yu Wang, Qing-Hai Ji, Dong-Mei Ji, Yu-Long Wang

**Affiliations:** 1 Department of Head and Neck Surgery, Fudan University Shanghai Cancer Center, Shanghai, China.; 2 Department of Oncology, Shanghai Medical College, Fudan University, Shanghai, China.; 3 Department of Clinical Oncology, The University of Hong Kong, Hong Kong, China.; 4 Department of Medical Oncology, Fudan University Shanghai Cancer Center, Shanghai, China.

**Keywords:** Oncology, Immunotherapy, Thyroid disease

## Abstract

Both anaplastic thyroid cancer (ATC) and papillary thyroid cancer (PTC) originate from thyroid follicular epithelial cells, but ATC has a significantly worse prognosis and shows resistance to conventional therapies. However, clinical trials found that immunotherapy works better in ATC than late-stage PTC. Here, we used single-cell RNA sequencing (scRNA-Seq) to generate a single-cell atlas of thyroid cancer. Differences in ATC and PTC tumor microenvironment components (including malignant cells, stromal cells, and immune cells) leading to the polarized prognoses were identified. Intriguingly, we found that CXCL13^+^ T lymphocytes were enriched in ATC samples and might promote the development of early tertiary lymphoid structure (TLS). Last, murine experiments and scRNA-Seq analysis of a treated patient’s tumor demonstrated that famitinib plus anti–PD-1 antibody could advance TLS in thyroid cancer. We displayed the cellular landscape of ATC and PTC, finding that CXCL13^+^ T cells and early TLS might make ATC more sensitive to immunotherapy.

## Abstract 2

Key differences in the tumor microenvironments of anaplastic thyroid cancer and papillary thyroid cancer may underlie the disparity in prognosis and sensitivity to immunotherapy observed in these cancers.

## Graphical abstract

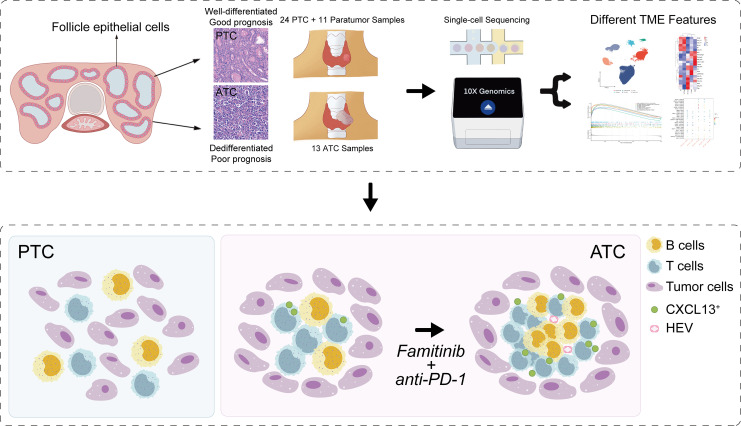

## Introduction

Thyroid cancer is the most prevalent endocrine malignancy, causing more than 40,000 deaths per year (1). Most thyroid cancers are thought to be pathologically derived from follicular epithelial cells, including papillary thyroid cancer (PTC) and anaplastic thyroid cancer (ATC). Although both PTC and ATC share a common origin, they have polarized clinical outcomes. PTC, accounting for nearly 80% of all thyroid cancers, has a favorable prognosis, with a 10-year disease-specific survival of over 90% (2). However, ATC is broadly regarded as one of the most lethal cancers, with a median survival of only 6 to 8 months (3), and ATC exhibits resistance to most conventional therapies (4).

Immunotherapy, such as immune checkpoint inhibitors (ICIs), is an anticancer treatment that harnesses modulated patient immune cells to treat multiple solid tumors (5). Recently, clinical trials have also applied ICIs in the management of treatment-refractory thyroid cancer, including ATC and late-stage PTC (6–8). Although ICI therapy might be a promising treatment for patients with aggressive thyroid cancers, distinct performances among different types of thyroid cancer have been revealed, with an overall response rate (ORR) of less than 30%. Notably, ATC was more responsive to anti–programmed cell death 1/programmed cell death ligand 1 (anti–PD-1/PD-L1) immunotherapy, while advanced PTC seemed to show a poorer response rate (9). However, the possible mechanisms through which immunotherapy is more effective for ATC than for PTC, and the ways to further enhance this efficacy, remain unclear, both of which are important directions to be explored in the future.

Recent advances in single-cell RNA sequencing (scRNA-Seq) have provided a new approach for profiling transcriptional features at cellular resolution in cancer. ScRNA-Seq has also been suggested as a promising avenue for identifying individuals who are candidates for precision medicine treatments such as immunotherapy (10). In our study, scRNA-Seq data comprising 226,066 cells from 21 patients with PTC and 13 patients with ATC were analyzed to profile the tumor microenvironment (TME) of PTC and ATC, the results of which represent the largest single-cell atlas of thyroid cancer to date. On this basis, we focused on the following aspects to explore the clinical questions mentioned above. First, we comprehensively displayed the cellular heterogeneity between PTC and ATC, exploring why ATC shows a more malignant phenotype than PTC. Second, we probed some potential key immune differences between the 2 kinds of thyroid cancer, which might determine their different responses to immunotherapy. Third, based on these findings combined with our constructed murine xenograft model and clinical trial results, we elucidated potential therapeutic strategies to improve immunotherapy for thyroid cancer.

## Results

### Single-cell expression atlas of PTC and ATC.

**Figure 1:**
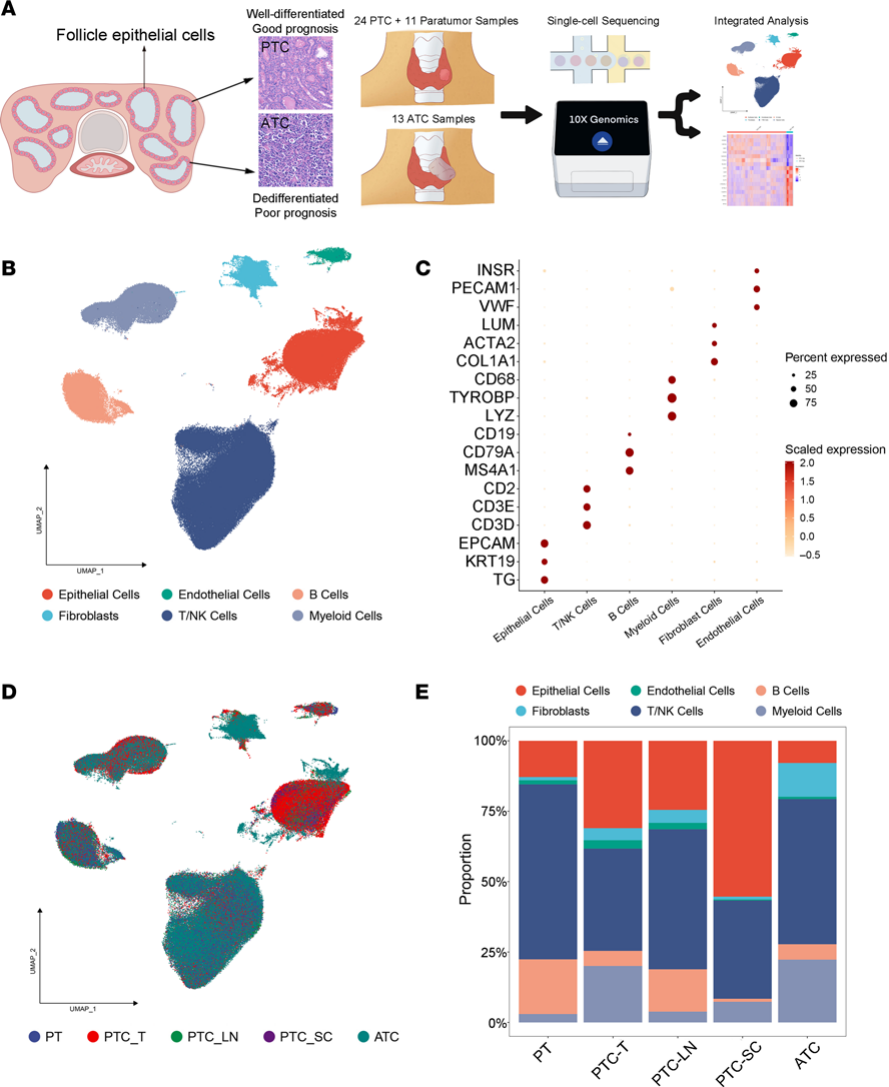
Overview of the TME in PTC and ATC at single-cell resolution. (**A**) Workflow of the sample collection and analysis process of the present study. (**B**) Uniform manifold approximation and projection (UMAP) plot of the quantified 226,066 cells categorized into 6 major clusters. (**C**) The expression of classic markers for each major cell lineage. (**D**) UMAP plot of the quantified cells colored by tissue type. PT, para-tumor; PTC_T, primary tumor of PTC; PTC_LN, lymph node metastasis of PTC; PTC_SC, subcutaneous loci of PTC; ATC, primary tumor of ATC. (**E**) Bar plot showing the percentage of each major cell lineage in different types of tissues.

We performed scRNA-Seq analysis to profile the cellular landscape of 48 samples from 34 patients with PTC or ATC, including samples of para-tumor tissues, primary tumors, lymph node (LN) metastases, and subcutaneous metastases (Figure 1A). Detailed information on these patients is shown in Supplemental Table 1; supplemental material available online with this article; https://doi.org/10.1172/jci.insight.173712DS1 After quality filtering and standard data processing, we identified a total of 226,066 single cells from the ATC and PTC patient samples. No profound batch effects of cell dissociation were noted (Supplemental Figure 1A). Subsequently, unsupervised clustering was performed, and we first annotated these cell clusters through the average expression of curated gene sets to identify major immune cell clusters (myeloid, T/natural killer [NK], and B cells), stromal cell clusters (endothelial cells [ECs] and fibroblasts), and epithelial cell lineages (Figure 1, B and C; Supplemental Figure 1B; and Supplemental Table 2). Of note, the cell type composition of individual tumors was also substantially varied among the 5 kinds of tissues (Figure 1, D and E) and 48 samples (Supplemental Figure 1C), indicating obvious intratumor heterogeneity, which was valuable to analyze.

### Different molecular characteristics of malignant epithelial cells from ATC and PTC.

**Figure 2:**
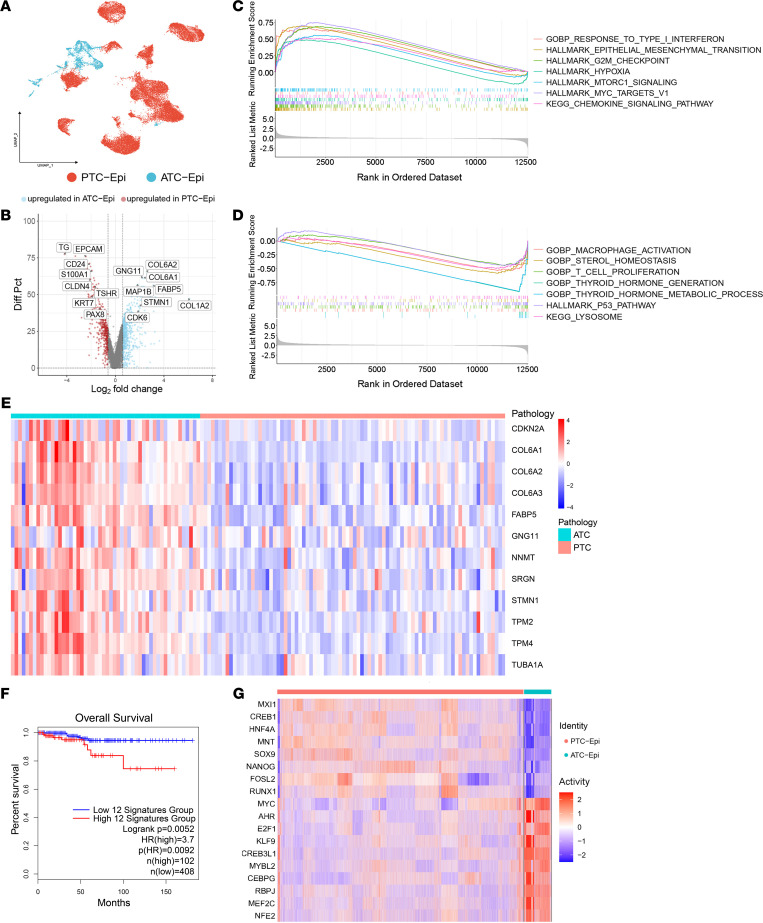
Distinct molecular features of ATC-Epis might contribute to the progression of ATC. (**A**) UMAP plot revealed 2 major subclusters of Epis from thyroid cancer: ATC-Epis and PTC-Epis. (**B**) Volcano plot showing the differentially expressed genes (DEGs) between ATC-Epis and PTC-Epis. Diff.pct, different percentage. (**C** and **D**) GSEA results of DEGs between ATC-Epis and PTC-Epis. (**E**) Heatmap of the 12-gene signature expression in public microarray data sets of PTC and ATC. (**F**) The 12-gene signature could predict poor clinical outcomes in TCGA-THCA cohort. (**G**) Heatmap of key transcription factors of ATC-Epis and PTC-Epis.

We first investigated the heterogeneity of malignant cells between thyroid cancer types by performing a similar unsupervised clustering analysis on epithelial cells from primary tumor samples (Supplemental Figure 2A). Significant heterogeneity of tumor cells was observed among these patients (Supplemental Figure 2A), which was similar to other scRNA-Seq tumor studies (11, 12). Finally, we defined 2 major epithelial lineages, ATC-Epis and PTC-Epis, separately, referring to their tissue origins (Figure 2A and Supplemental Figure 2, B and C). A common marker gene, *TMSB4X*, of PTC-Epis and ATC-Epis was also discovered, implying this was a potential novel biomarker for ATC and PTC (Supplemental Figure 3).

Next, we compared the differentially expressed genes (DEGs) between ATC-Epis and PTC-Epis. Thyroid differentiation-related genes (*TG*, *TSHR*, and *PAX8*) were upregulated in PTC-Epis and downregulated in ATC-Epis, which revealed a potential dedifferentiation process in thyroid cancer progression (Figure 2B, Supplemental Figure 2C, and Supplemental Table 3) (13). However, ATC-Epis showed high expression of invasion-related genes (*COL6A2*, *GNG11*, and *MAP1B*) and cell proliferation markers (*STMN1* and *CDK6*) (Figure 2B) (14–16). Moreover, monocle3 trajectory analysis also revealed the underlying dedifferentiation process from PTC to ATC (Supplemental Figure 4), though the continuous process should be confirmed experimentally. Additionally, gene set enrichment analysis (GSEA) showed that epithelial-mesenchymal transition (EMT) and some other procancer pathways were activated in ATC-Epis (Figure 2C). In contrast, thyroid function-related pathways were downregulated in ATC-Epis (Figure 2D).

Moreover, based on the top DEGs of ATC-Epis, we established a 12-gene ATC signature to distinguish PTC and ATC containing multiple EMT markers. The signature was further validated in public microarray data (Figure 2E). This gene signature was also shown to predict a poor prognosis in The Cancer Genome Atlas-Thyroid Cancer (TCGA-THCA) data set (Figure 2F). In addition, regulon analysis further revealed the stemness of ATC-Epis. The activation of cell cycle–related transcription factors (TFs) (*E2F1*, *CEBPG*, and *MYC*) and TFs related to thyroid differentiation and regeneration (*SOX9* and *NANOG*) was dominant in ATC-Epis (Figure 2G) (17, 18). Together, these results showed that ATC-Epis had fatal, dedifferentiated, and stem-like features compared with PTC-Epis.

### A refined classification of the tumor stroma revealed a potentially novel subgroup of ATC cancer-associated fibroblasts.

**Figure 3:**
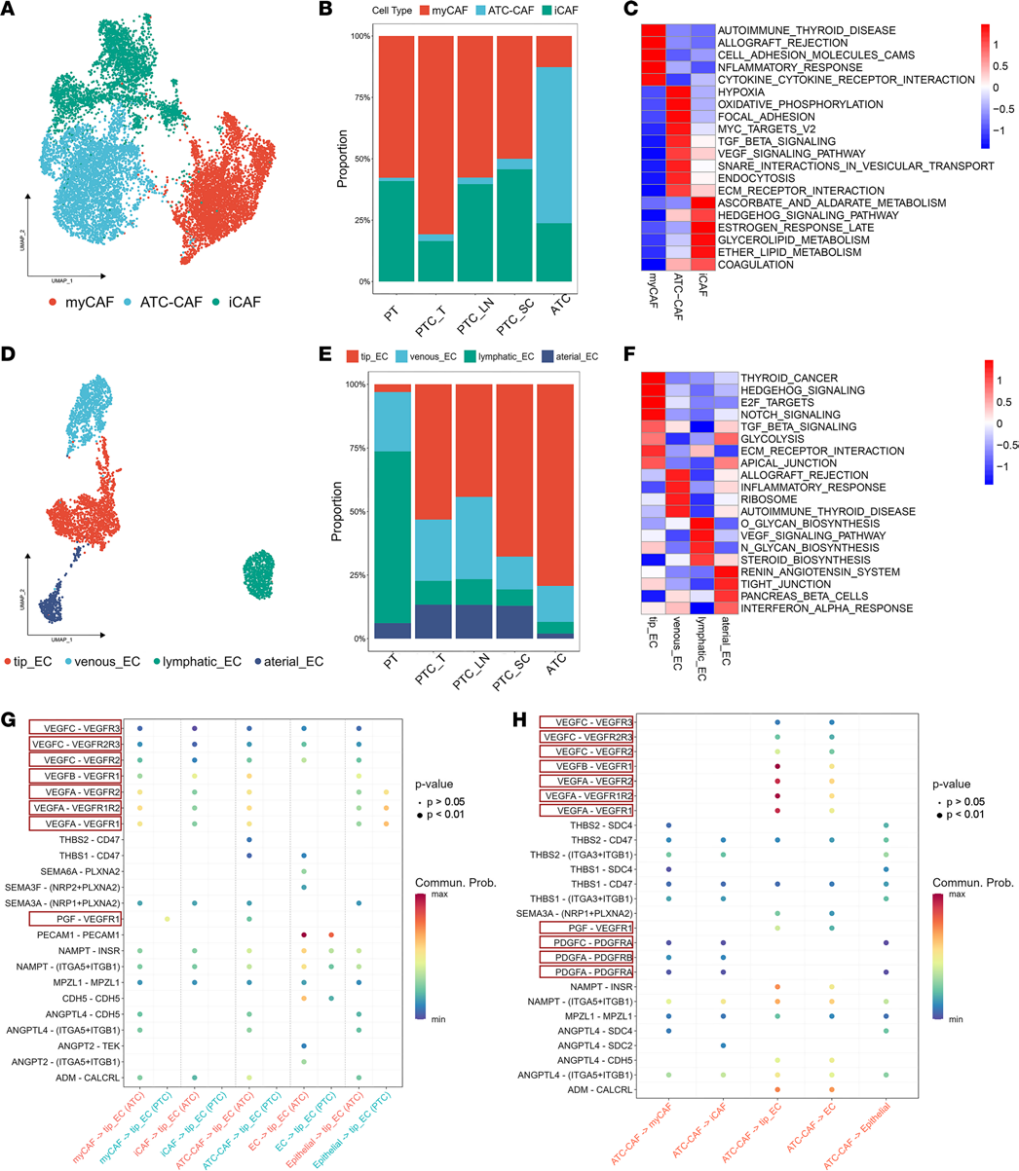
Tumor stromal dynamics of ATC and PTC. (**A**) UMAP plot depicting 3 clusters of cancer-associated fibroblasts (CAFs) in ATC and PTC. (**B**) Bar plot showing the percentage of each CAF subcluster in different types of tissues. (**C**) Heatmap of GSVA results of upregulated genes of 3 CAF subclusters. (**D**) UMAP plot depicting 4 clusters of endothelial cells (ECs) in ATC and PTC. (**E**) Bar plot showing the percentage of each CAF subcluster in different types of tissues. (**F**) Heatmap of GSVA results of upregulated genes of 4 EC subclusters. (**G**) Different cell-cell interaction patterns between Epis or stromal cells and tip_EC in ATC and PTC samples. Key ligand-receptor (L-R) pairs are highlighted with red boxes. Commun. Prob., communication probability. (**H**) Bubble plot showing the potential L-R pairs between epithelial cells or stromal cells and ATC-CAFs. Key L-R pairs are highlighted with red boxes.

To study stromal components in the TME, we first identified 16,472 presumed cancer-associated fibroblasts (CAFs) and ECs, as shown in Figure 1A. The subclustering of fibroblasts revealed 3 distinct clusters: 2 known fibroblast subgroups, including myofibroblastic CAFs (myCAFs) and inflammatory CAFs (iCAFs), and a potentially novel subgroup named ATC-CAFs (Figure 3A and Supplemental Figure 5A). The markers of each cell lineage are displayed in Supplemental Figure 3A. iCAFs and myCAFs comprised the main fibroblast types in normal tissue and PTC tumor samples, respectively. However, the subgroup of ATC-CAFs were mainly present in ATC (63.6%) (Figure 3B). ATC-CAFs showed high expression levels of genes associated with tumor invasion (*MMP14*, *LOXL2*, and *PGK1*) (19, 20). Gene set variation analysis (GSVA) further revealed that hypoxia and extracellular matrix (ECM) receptor interaction pathways were enriched in ATC-CAFs, suggesting a procancer role played by ATC-CAFs in the TME (Figure 3C).

Refined subclustering of ECs identified 4 clusters of ECs called tip ECs, lymphatic ECs, venous ECs, and arterial ECs (Figure 3D and Supplemental Figure 5B) (21). We noticed that tip ECs occupied a larger percentage of tumor tissues, especially in ATC tumor samples (79.3%) (Figure 3E). Pathway enrichment analysis showed that glycolysis and thyroid cancer–related pathways were activated in tip cells, which implied an abnormal state of tumor vessels (22) (Figure 3F). Intriguingly, the ECM receptor pathway was also enriched in ATC-enriched tip ECs, such as ATC-CAFs, indicating that the ECM played an important role in thyroid cancer progression.

Previous studies also revealed that the ECM could contribute to tumor-cell interactions (23), and we thus evaluated cell-cell communication among tip ECs, ATC-CAFs, and malignant cells. We observed multiple VEGF-VEGFR ligand-receptor (L-R) pairs involved in cell-cell interactions with tip cells in thyroid cancer, especially in ATC (Figure 3G). We also showed that ATC-CAFs could contribute to the reeducation of cancer cells on invasion and proliferation by PDGF and VEGF L-R pairs (Figure 3H) (24). Notably, Hashimoto’s thyroiditis (HT) also had an impact on the epithelial-stromal interaction (25). To explore the distinguishing epithelial-stromal interaction dynamics between HT and thyroid carcinoma, we downloaded the scRNA-Seq data of patients with HT reported by Zhang et al. (26). We identified different cell lineages and found the complex cell-cell communication pattern between thyrocytes and stromal cells in HT (Supplemental Figure 6, A and B). That further verified the specific procancer role of tumor stroma in ATC. According to GSEA and cell-cell interaction patterns, cells of the tumor stroma, especially tip ECs and ATC-CAFs, could play a role in ATC invasiveness.

### The more suppressive immune microenvironment in ATC compared with PTC.

**Figure 4:**
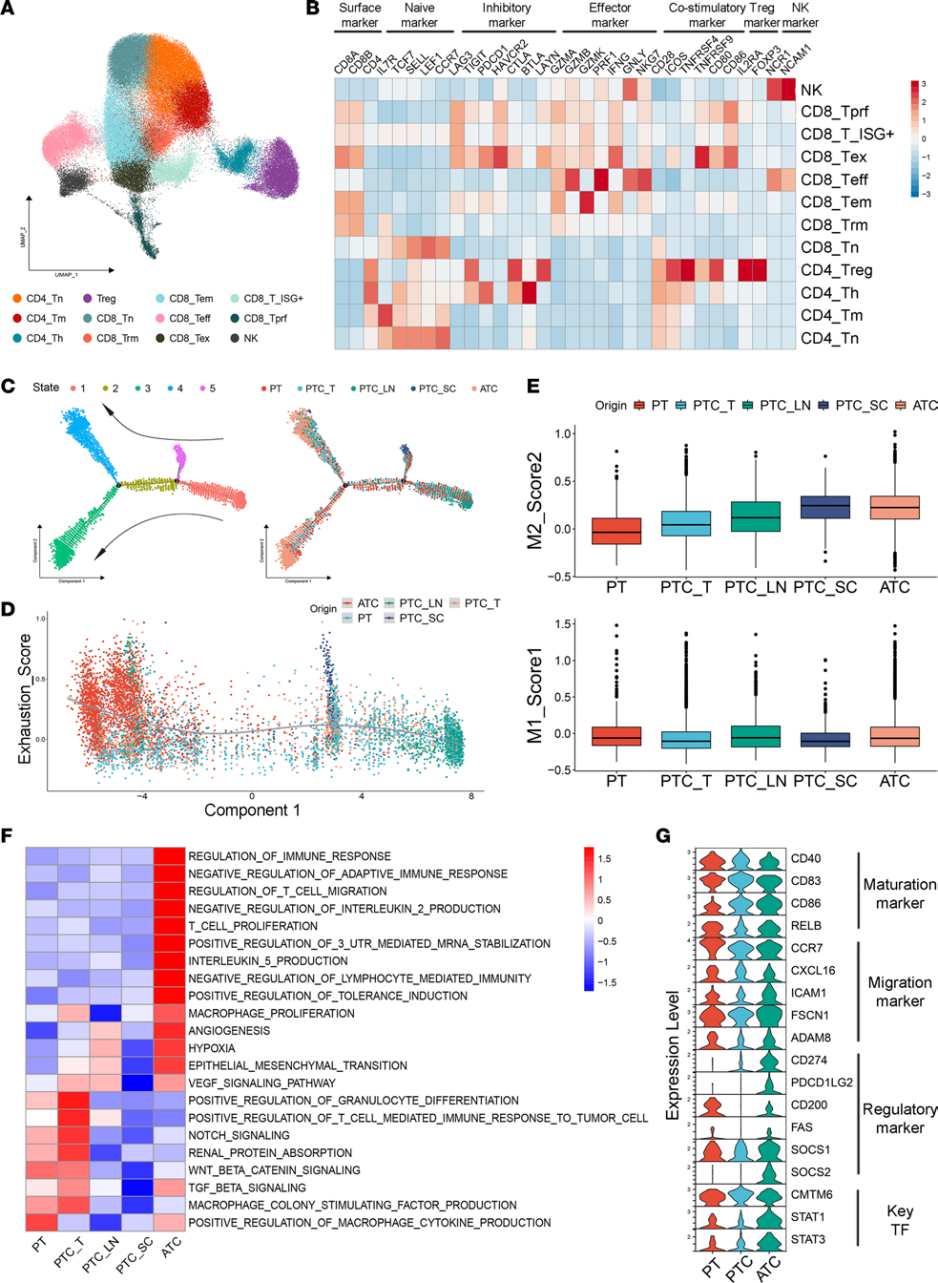
Multiple kinds of immune cells form a more immunosuppressive phenotype in ATC than in PTC. (**A**) UMAP plot depicting 12 subgroups of T and NK lymphocytes from ATC and PTC samples. (**B**) Heatmap of the expression of canonical functional markers among 12 kinds of T or NK cells. (**C**) Trajectory analysis revealed distinct cell states of CD8^+^ T cells from ATC and PTC samples. (**D**) Correlation between calculated Exhaustion_Score and Component 1. T lymphocytes were stained according to their tissue origin. (**E**) M1 and M2 scores of macrophages from different tissues. (**F**) Heatmap of GSVA results of upregulated genes in macrophages from different tissues. (**G**) Violin plots of key DC-related biomarker expression in LAMP3-DCs derived from PT, PTC, and ATC samples.

Our dissection of PTC and ATC immune components showed that T/NK cells were the most prevalent cell type in thyroid cancer (Figure 1C). According to the classic functional markers of different T cell subclusters (27), we identified 12 clusters: CD4^+^ naive T (CD4 Tn), CD4^+^ T helper (CD4_Th), CD4^+^ memory T (CD4_Tm), regulatory T (Treg), CD8^+^ naive T (CD8_Tn), CD8^+^ resident memory T (CD8_Trm), CD8^+^ effector memory T (CD8_Tem), CD8^+^ effector T (CD8_Teff), CD8^+^ exhausted T (CD8_Tex), ISG-positive CD8^+^ T (CD8_T_ISG+), CD8^+^ proliferating T (CD8_Tprf), and NK cells (Figure 4, A and B, and Supplemental Figure 7A). Interestingly, the percentage of T cells that expressed exhaustion markers (CD8_Tex, CD8_Tprf, and CD8_T_ISG+ cells) was significantly higher in ATC samples than in PTC primary tumor samples (Supplemental Figure 7, B and C). In contrast, cytotoxic cells (CD8_Teff and NK cells) appeared more frequently in PTC than in ATC (Supplemental Figure 7C).

Next, trajectory analysis was applied to the evolutionary dynamics of CD8^+^ T cells, and we found that naive T cells from LNs were located at the starting state, while T cells from ATC were at the endpoint (Figure 4C and Supplemental Figure 7D). Furthermore, a terminally differentiated and more exhausted state of T cells exhibited dominance in ATC, as verified by the correlation analysis between Monocle Component 1 and the score of the exhausted T cell signature (Figure 4D) (28).

Then, we studied myeloid cells of thyroid cancer and obtained 6 subclusters: macrophages, monocytes, type 1 and type 2 conventional dendritic cells, plasmacytoid dendritic cells, and LAMP3-DCs (Supplemental Figure 8). First, an M2 phenotype of macrophages in ATC was noted, which indicated that M2 tumor-associated macrophages (TAMs) in ATC might contribute to the suppressive immune microenvironment in ATC (Figure 4E). GSVA further demonstrated that TAMs could play a procancer role in ATC in multiple ways, including through immune processes, angiogenesis processes, and hypoxia reactions (Figure 4F). Second, for dendritic cells (DCs), we also identified a mature regulatory DC subgroup named LAMP3-DCs, which expressed multiple immune regulatory markers (Supplemental Figure 9). These immunoregulatory molecules were mainly derived from ATC-derived LAMP3-DCs (PD-L1 and PD-L2) (Figure 4G), and the upregulation of key TFs (*STAT1* and *STAT3*) could partially explain the enhanced expression of PD-L1 (Figure 4G). That implied the potential therapeutic value of targeting LAMP3-DCs, which was valuable to explore in more detail. Overall, the landscape of T/NK cells and myeloid cells revealed an immunosuppressive TME in ATC.

To establish a comprehensive overview of the immune TME of thyroid cancer, we also studied the B cell components of thyroid cancer and identified 4 clusters, including germinal center B (GC B) cells, naive B cells, memory B cells, and plasma cells (Supplemental Figure 10, A and B). The composition of each B cluster varied among the 5 tissue types (Supplemental Figure 10C). Interestingly, GC B cells and plasma cells occupied a larger proportion in ATC, while common B cells (naive B cells and memory B cells) were dominant in PTC tissue.

### Subgroup identification of CXCL13^+^ T cells in ATC.

**Figure 5:**
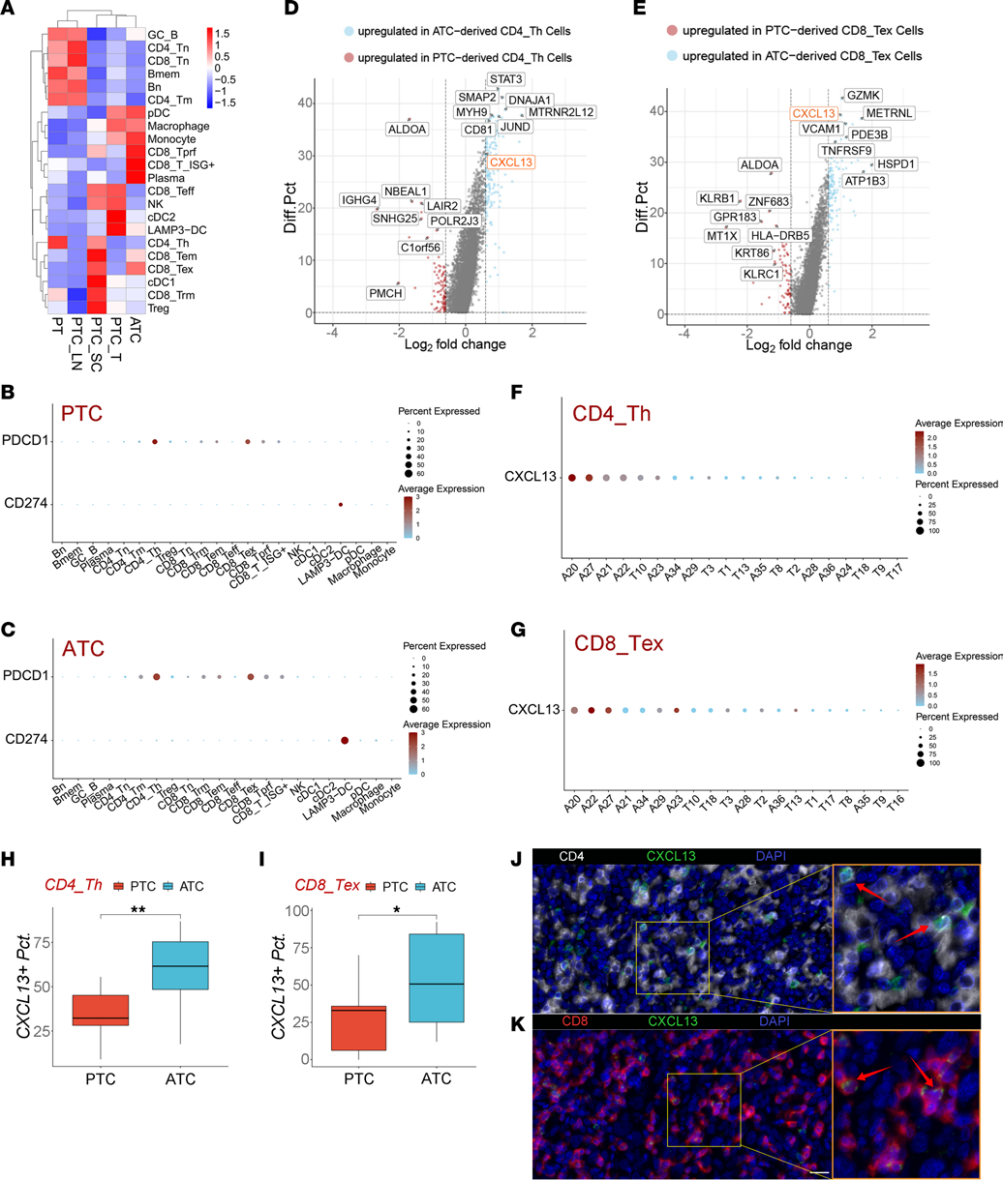
Existence of CXCL13^+^ exhausted T cells in ATC tumors. (**A**) Tissue preference of different immune cell subclusters evaluated by R/oe. (**B** and **C**) PDCD1 and PD-L1 expression in the tumor immune microenvironment of PTC (**B**) and ATC (**C**). (**D**) Volcano plot of DEGs between ATC-derived CD4_Th cells and PTC-derived CD4_Th cells. *CXCL13* was upregulated in ATC-derived cells. (**E**) Volcano plot of DEGs between ATC-derived CD8_Tex cells and PTC-derived CD8_Tex cells. *CXCL13* was upregulated in ATC-derived cells. (**F** and **G**) Dot plots of *CXCL13* expression in CD4_Th (**F**) or CD8_Tex (**G**) cells from different patients. Patients who contained fewer than 50 single cells that were identified as CD4_Th or CD8_Tex were removed from these plots. (**H** and **I**) In the CD4_Th (**H**) or CD8_Tex (**I**) subpopulation, the percentage of CXCL13^+^ cells was significantly higher in ATC primary tumors than in PTC primary tumors. Box plots show the interquartile range, median (line), and minimum and maximum (whiskers). Statistical analysis: Student’s 2-tailed *t* test (*: *P* < 0.05, **: *P* < 0.01, ***: *P* < 0.001). (**J**) Representative mIHC image showing the coexpression of CD4 (CD4^+^ T cells, white) and CXCL13 (green) in an ATC section, with nuclei stained by DAPI (blue). CD4^+^CXCL13^+^ T lymphocytes are marked by red arrows. (**K**) Representative mIHC picture showing coexpression of CD8 (CD8^+^ T cell, red) and CXCL13 (green) in an ATC section, with nuclei stained by DAPI (blue). CD8^+^CXCL13^+^ T lymphocytes are marked by red arrows. Scale bar: 20 μm. Original magnification: left: 150×; right: 300×.

Next, we evaluated the tissue preference of each immune cell lineage by calculating the ratio of observed over expected cell numbers (R/oe) of each immune cell subgroup (Figure 5A) (29). An abnormal immune TME state existed in primary tumors and subcutaneous metastases, which dominantly contained myeloid cells and mature T cells. Another immune microenvironment state, characterized by enriched naive and memory cells, was found in normal thyroid gland tissues and LNs.

Of note, exhausted T cells (CD8_Tex, CD8_Tprf, and CD4_Th cells), which could be reactivated by ICIs, persisted in tumor samples and were more enriched in ATC samples (Figure 5A). We further observed a higher expression of PD-1 and PD-L1, the immune checkpoints, in ATC than in PTC and revealed that CD8_Tex and CD4_Th cells were the main origins of *PDCD1* mRNA expression (Figure 5, B and C).

Subsequently, the analysis of DEGs between ATC-derived and PTC-derived exhausted T cells was conducted, where we found some biomarkers related to T cell function were differentially expressed (30). Distinct molecular features of CD4_Th and CD8_Tex from PTC and ATC could also contribute to polarized clinical outcomes. Notably, the upregulation of *CXCL13* was identified in both CD4_Th and CD8_Tex cells from ATC samples (Figure 5, D and E, and Supplemental Tables 4 and 5). In addition, sample A20 in our study, from a patient who had previously received anti–PD-L1 therapy, contained the highest percentage of CXCL13^+^ T cells among both CD8_Tex and CD4_Th cells, suggesting that immunotherapy induced the expansion of CXCL13^+^ T cells (Figure 5, F and G). The proportions of CXCL13^+^ CD8_Tex and CD4_Th cells in ATC samples were also significantly higher than those in PTC samples (Figure 5, H and I). Microarray data of thyroid cancer verified that the expression of *CXCL13* was significantly higher in ATC than poorly differentiated thyroid cancer (PDTC) and PTC (Supplemental Figure 11). In addition, the multicolor IHC (mIHC) images further validated that CXCL13^+^CD8^+^ T cells and CXCL13^+^CD4^+^ T cells existed extensively in ATC specimens (Figure 5, J and K). These findings denoted a potentially novel subgroup of CXCL13^+^CD8^+^ and CXCL13^+^CD4^+^ T cells that existed in ATC but not PTC.

### Formation of early TLSs in ATC.

**Figure 6:**
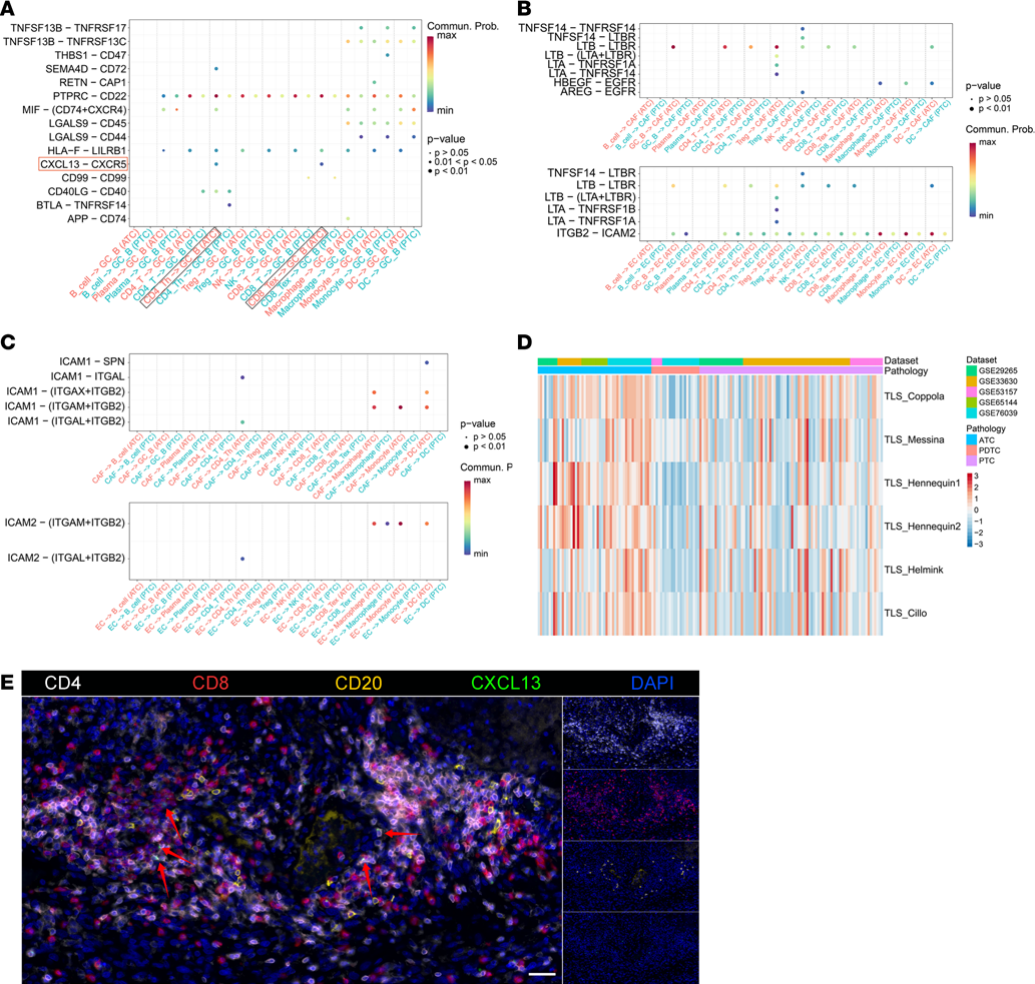
Potential early TLSs characterized in ATC samples. (**A**) Different cell-cell interactions between GC B cells and immune cell subclusters between ATC and PTC. A key L-R pair (CXCL13-CXCR5) was identified between CD4_Th, or CD8_Tex, and GC B cells in ATC but not in PTC. (**B**) Lymphotoxin signaling cell-cell communication was upregulated in ATC. (**C**) Adhesion molecule–involved cell-cell communications were upregulated in ATC. (**D**) Heatmap of previously reported TLS signature scores in public ATC and PTC microarray data. (**E**) Representative mIHC image showing the coexpression of CD4 (CD4^+^ T cells, white), CD8 (CD8^+^ T cells, red), CD20 (B cells, yellow), and CXCL13 (green) in early TLSs in an ATC section. CXCL13^+^ T lymphocytes are marked by red arrows. Scale bar: 50 μm. Original magnification: left: 100×; right: 25×.

To investigate the role of CXCL13**^+^** T lymphocytes in the TME, we conducted cell-cell interaction analysis among immune cell clusters. The results showed that GC_B cells could be recruited by the CXCL13-CXCR5 interaction from CD8_Tex and CD4_Th cells in ATC, implying the potential development of early tertiary lymphoid structures (TLSs) in ATC (Figure 6A). This result also partially explained the reason for the highest GC_B cell infiltration in ATC among all tumor samples (Figure 5A). To verify potential TLS development, we explored more molecular inducers of TLSs in ATC and PTC. We found dense lymphotoxin beta/lymphotoxin beta receptor communication between various immune cells and CAFs in ATC but not in PTC (Figure 6B). As Figure 6C shows, adhesion molecules could also recruit lymphocytes into the ATC tumor, which has been reported to contribute to TLS formation (31). Overall, the communication niche favored a complex TME to support the development of TLSs in ATC.

Additionally, TLSs tended to develop in ATC tumors rather than PTC or PDTC tumors, depending on previously reported TLS gene signatures and CXCL13 expression (Figure 6D, Supplemental Figure 11, and Supplemental Table 6) (32–35). Therefore, we deduced the deficiency of TLSs in PDTC. The PDTC-related findings also echoed an immune-exclusive phenotype identified in PDTC previously (36). Moreover, using mIHC technology, we also directly detected the spatial distributions of lymphocytes in ATC. The coexistence of T and B cell aggregates was found in a section from an ATC patient without HT (Figure 6E), illustrating early immature TLS formation in ATC (37). Finally, CXCL13^+^ T cells were also observed in these immature TLSs, suggesting a potential role of CXCL13^+^ T cells in recruiting B cells and initiating the development of immature TLSs (Figure 6E).

Previous studies also identified TLSs in HT (26, 38). To provide more details on potential TLSs in HT, we reevaluated the cellular crosstalk in HT based on scRNA-Seq data analysis. Interestingly, we observed that chemokine-positive stromal cells, rather than CXCL13^+^ T cells in ATC, played a central role in TLS development in HT, which could enlist GC B cells into TLSs in HT (Supplemental Figure 12A). Also, CXCL12/CXCR4 could be the crucial axis for recruiting T cells and B cells and TLS development in HT (Supplemental Figure 12B), which was different from early TLS formation in ATC. Furthermore, by integrating analyses, we also directly verified the CXCL12 dominance in the HT-derived stromal cells, rather than cells from normal tissue or HT-free thyroid cancer samples (Supplemental Figure 12, C and D).

### Famitinib enhances immunotherapeutic efficacy by improving immune infiltration and TLS development in murine cancer models.

**Figure 7:**
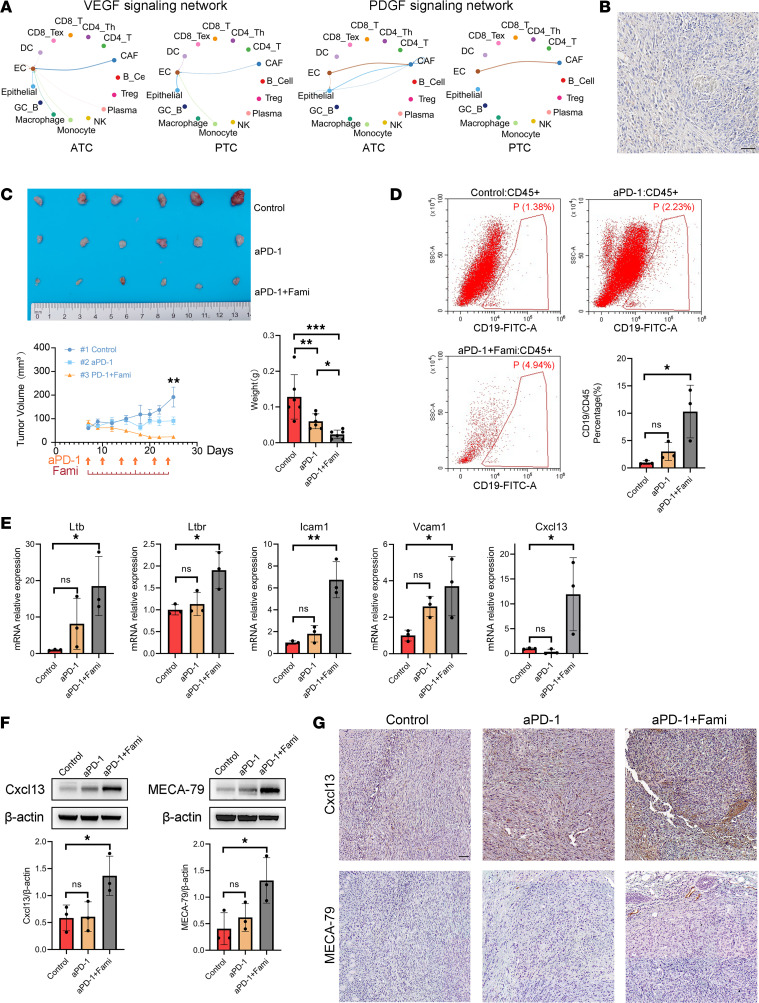
Famitinib plus immunotherapy could improve the antitumor effect in a murine thyroid cancer model by creating a pro-TLS microenvironment. (**A**) VEGF signaling and PDGF signaling cell-cell communications were upregulated in ATC rather than PTC and involved with ECs. (**B**) IHC image showed nearly negative expression of the HEV marker (MECA-79) around the early TLSs identified in Figure 6E. Scale bar: 50 μm. (**C**) Tumor images, growth curve, and weight of the control group, anti–PD-1 (aPD-1) group, and famitinib plus aPD-1 (aPD-1+Fami) group of BPC xenografts. Statistical analysis: 1-way ANOVA followed by 2-stage step-up method of Benjamini, Krieger, and Yekutieli FDR procedure (*: *q* < 0.05, **: *q* < 0.001, ***: *q* < 0.001). *n* = 6 per group. (**D**) Representative flow cytometry images of each group of xenografts and the bar plot show an increase in infiltrated CD19^+^ B cells in the aPD-1+Fami group. Statistical analysis: 1-way ANOVA followed by Tukey multiple-comparison test or Kruskal-Wallis test followed by Dunn’s multiple comparisons test (*: *P* < 0.05). *n* = 3 per group. (**E**) QPCR results indicated higher mRNA expression of biomarkers related to TLS development in the aPD-1+Fami group (*Ltb*, *Ltbr*, *Icam1*, *Vcam1*, and *Cxcl13*). Statistical analysis: 1-way ANOVA followed by Tukey multiple-comparison test (*: *P* < 0.05, **: *P* < 0.01). *n* = 3 per group. (**F**) Western blot images and histograms suggested upregulation of Cxcl13 and MECA-79 in the aPD-1+Fami group. Statistical analysis: 1-way ANOVA followed by Tukey multiple-comparison test (*: *P* < 0.05). *n* = 3 per group. (**G**) Representative IHC staining of Cxcl13 and MECA-79 showed that both markers were upregulated in the aPD-1+Fami group. Scale bar: 50 μm.

Our study identified early TLSs in ATC tumors. Although the ORR to immunotherapy in patients with ATC is still low, it could be promising to promote the number and maturation of TLSs in ATC to improve the effect of immunotherapy. Aberrant tumor vessels hampered TLS formation (39), and more severe tumor vascular abnormalities led by VEGF/PDGF signaling were identified in ATC than in PTC (Figure 3G and Figure 7A). Moreover, we also found a lack of high endothelial vessels (HEVs) in sections from patients with early TLS-positive ATC (Figure 7B), while HEVs could recruit key immune cells such as CXCL13^+^ T cells into advanced TLSs (40).

To reverse this, famitinib, a novel anticancer drug targeting VEGF/PDGF signaling, was added to conventional anti–PD-1 (aPD-1) immunotherapy to investigate its role in treatment effect improvement. First, *Braf^CA/+^ Trp53^fl/fl^ Tpo-CreER^T2^* transgenic mice were constructed, and we successfully isolated an immortalized primary thyroid cancer cell line (Supplemental Figure 13) (41). Tumors were induced in the thyroids of the transgenic mice (Supplemental Figure 13B), and we further verified these tumors were murine primary thyroid cancer by hematoxylin-eosin (HE) staining and IHC (Supplemental Figure 13C). Next, the allograft of the BPC cell line also presented similar histopathological features to primary ATC, which provided evidence that the BPC xenograft could simulate ATC models (Supplemental Figure 13D).

Later, BPC allografts were constructed and treated with drugs, including famitinib and aPD-1 antibody. Mice treated with the combination of aPD-1 and famitinib showed significantly suppressed tumor growth compared with single-agent aPD-1 therapy (Figure 7C). Flow cytometry data showed that the addition of famitinib enhanced the infiltration of B cells in the murine thyroid cancer model, indicating that famitinib plus aPD-1 may be helpful in promoting TLS formation (Figure 7D). Next, quantitative PCR (qPCR) results showed that several key molecules involved in TLS development were upregulated in the combined therapy group, including *Ltb*, *Ltbr*, *Icam1*, *Vcam1*, and *Cxcl13* (Figure 7E) (42). Finally, by Western blot and IHC, we further verified the upregulation of Cxcl13 and MECA-79 protein (HEV markers) after mice received aPD-1 plus famitinib combined therapy, which implied the formation of HEVs and latent induction of TLSs (Figure 7, F and G).

Furthermore, the combination therapy could also reshape the microenvironment of MC38 tumor xenografts to potentially induce TLSs by enhancing B cell infiltration (Supplemental Figure 14). However, only some of the TLS-related biomarkers were upregulated (Supplemental Figure 14D). In all murine experiments, famitinib and immunotherapy had a positive synergistic effect, as famitinib created a pro-TLS microenvironment in various solid tumors, but it might perform better in thyroid carcinoma.

### Famitinib plus camrelizumab reshapes the TME and promotes TLS development in a patient with ATC.

To further evaluate the performance of the combined therapy in clinical use, we are also conducting a registered clinical trial assessing the intervention of famitinib plus camrelizumab (a commercial aPD-1 antibody) in the late stage of thyroid cancer (ClinicalTrials.gov NCT04521348). The combined therapy has displayed good efficacy in ATC. Some patients with unresectable tumors have achieved a partial response (PR) after several therapeutic cycles, which could allow them to undergo subsequent operations.

**Figure 8:**
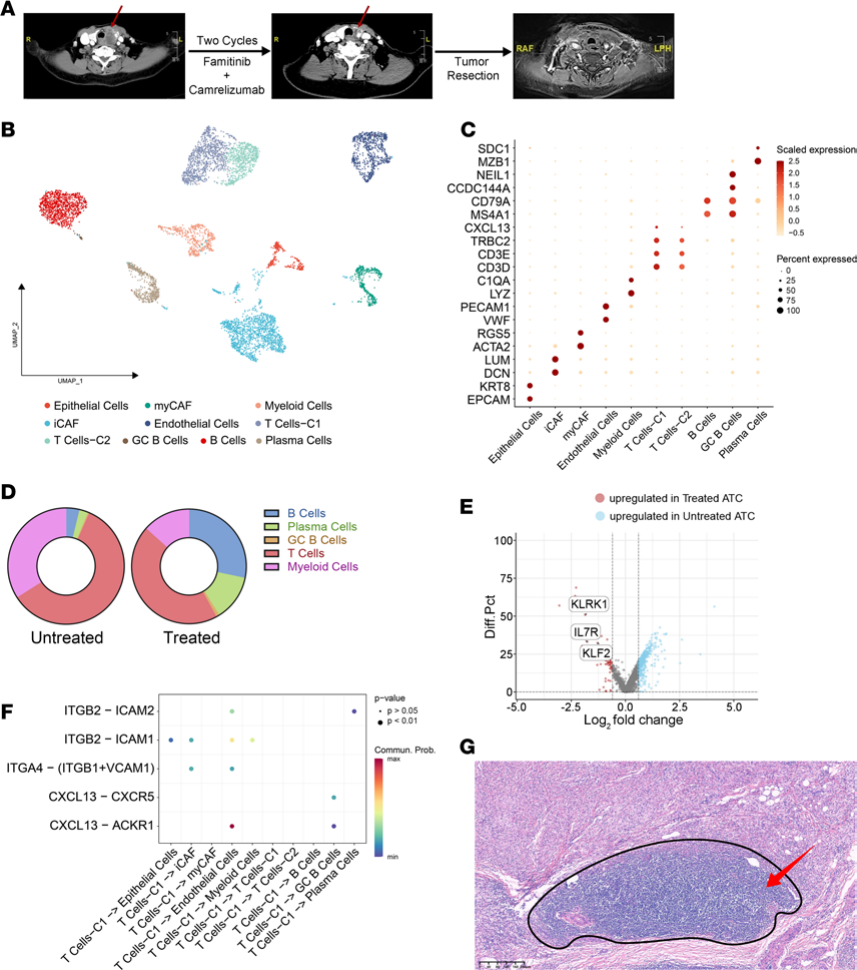
The pro-TLS cellular landscape of a patient with ATC treated with famitinib plus camrelizumab. (**A**) Neck CT and MRI images of a woman with ATC (A37) receiving famitinib + camrelizumab therapy and surgery at different time points during her treatment. The primary thyroid tumor is marked by a red arrow. RAF, right anterior foot; LPH, left posterior head. (**B**) UMAP plot of cells from the famitinib + camrelizumab-treated patient showed 10 major cell lineages. (**C**) Bubble plot of marker genes of each cell cluster. (**D**) Pie charts of immune components of untreated and treated patients with ATC showed an increased percentage of B lymphocytes (B cells, plasma cells, and GC B cells) in treated patients. (**E**) Volcano plots of DEGs between *CXCL13*-high T cells from treated patients (T Cells-C1) and untreated patients (CD4_Th and CD8_Tex). (**F**) Cell-cell communication pattern of TLS-related signaling between *CXCL13*-high T cells (T Cells-C1) and other cell clusters in the treated patient. (**G**) HE staining image suggested TLS development in a patient with ATC treated with famitinib + camrelizumab. Scale bar: 200 µm.

For instance, a 74-year-old, HT-free woman was diagnosed with ATC by pathologists and enrolled in our clinical trial. We observed a PR after 2 cycles of combined therapy, and then she underwent tumor resection (Figure 8A). Subsequently, we conducted scRNA-Seq analysis of the postoperative tumor specimen from this patient, and 10 major cell clusters were identified, including epithelial cells, iCAFs, myCAFs, ECs, myeloid cells, common B cells, GC B cells, plasma cells, and 2 groups of T lymphocytes (Figure 8, B and C). Consistent with the xenograft flow cytometry results, we also found greater infiltration of B lymphocytes (B cells, plasma cells, and GC B cells) in this sample than in the treatment-naive ATC samples analyzed before (Figure 8D). Moreover, we also identified a cluster of T cells (T cells-C1) with high *CXCL13* expression in this patient (Figure 8C). In addition, several key functional genes (*IL7R*, *KLRK1*, and *KLF2*), which are important for the maintenance of memory T cells (43, 44), were upregulated in CXCL13^+^ T cells derived from this treated patient (Figure 8E).

Furthermore, the CXCL13/CXCR5 axis was also identified to be a key L-R pair in the communication process of T cells-C1 and GC B cells, which also implied the potential development of TLSs in patients receiving combined therapy. In addition, therapy-induced, normalized tumor vessels could also recruit CXCL13^+^ T cells through CXCL13-ACKR1 or adhesion molecule–involved L-R pairs (Figure 8F). For other molecules involved in TLS development, we observed sustained expression in cells from this patient (Supplemental Figure 15). Strikingly, we found a large amount of lymphocyte infiltration and TLS formation in the patient’s postoperative pathological section (Figure 8G). This finding echoed the results of in vivo experiments on murine xenografts and further favored a potential combined therapeutic strategy that may help the development of TLSs and sensitization to ICIs.

## Discussion

Thyroid cancer, most of which is PTC, is the most common endocrinology malignancy, with a relatively favorable prognosis. However, ATC is a highly aggressive subtype of thyroid cancer with high morality because of the aggressive characteristics and a lack of effective treatments (45). Although both ATC and PTC originate from normal glandular epithelial cells, the molecular mechanism leading to the polarized clinical outcomes remains unclear (46). Immunotherapy is an anticancer treatment that has recently been used in thyroid cancer trials. The ORR to ICIs was higher in ATC than in PTC (9). However, the reason for the different sensitivities to immunotherapy between late-stage PTC and ATC remains unexplored. In the present study, we integrated and analyzed the transcriptomic features of 226,066 cells from patients with PTC or ATC, including samples from primary tumors, peripheral normal thyroid gland tissue, and metastatic loci. Notably, we have incorporated high-risk PTC subtype cases and a sample consisting of both PTC and ATC, indicating that the current integrated analysis would depict thyroid TME in detail.

First, to study the uncovered causes of the differences in mortality rates, we studied the invasive cancer cells of PTC and ATC. Using scRNA-Seq, we directly identified the distinct transcriptomes of malignant epithelial cells in ATC and PTC. We highlighted the central role of EMT in ATC-Epi invasiveness. Moreover, there are no generally accepted pathological markers for ATC diagnosis (47). Thus, we constructed a novel 12-gene signature based on scRNA-Seq analysis results, providing potential diagnostic value to distinguish ATC and PTC. Moreover, our results also revealed a potential dedifferentiation process of ATC malignant cells with trajectory analysis and DEG analysis, which lost thyroid-specific markers and gained stemness during cancer progression (16, 46, 48). In addition, cell-cell interaction patterns also highlighted the essential and reeducational role of cancer cells in the thyroid cancer microenvironment.

CAFs play an important role in cancer development. It was reported that myCAFs and iCAFs could promote cancer progression through distinct cell-cell communication niches (49). In addition to these 2 common CAF subpopulations, we identified a subgroup called ATC-CAFs specifically enriched in ATC stromal components, characterized by significantly upregulated ECM receptor signaling and dense cell-cell communication with various cells in the ATC TME (Figure 3F). Notably, Li et al. (50) identified a similar group of proinvasive CAFs named ECM CAFs in diffuse-type gastric cancer. The ATC-CAF subgroup could also lead to the formation of abnormal tumor veins through VEGF and PDGF interactions between aberrant tip ECs. These results implied that ATC-CAF–like fibroblasts might promote progression to the advanced stage in different cancers.

The immune TME, containing multiple tumor-infiltrating lymphocytes and suppressive myeloid cells, is essential for cancer escape activity (51). Compared with PTC, a more suppressive immuno-microenvironment was identified in ATC in this research. Both T cell exhaustion and M2 polarization of macrophages are dynamic processes contributing to tumor progression (52, 53). Our study revealed an enrichment of terminally differentiated exhausted T cells with higher *PDCD1* expression and found a cluster of M2-polarized macrophages in ATC primary tumors that could promote the process of immune escape. In addition, similar to the LAMP3-DCs identified in our study, Maier et al. found that mature DCs enriched in immunoregulatory molecules could limit anticancer immunity in human cancers and mouse models (54). Together, these findings helped us understand the contribution of the suppressive immune TME to the high lethality of ATC at the single-cell level. Furthermore, the results also implied that a more detailed prospective study of immunotherapy targeting PD-1/PD-L1 in ATC could be valuable.

Anti–PD-1/PD-L1 immunotherapy, which aims to reactivate exhausted T cells, has been used to successfully manage various advanced cancers (55, 56). Recently, some clinical trials also measured the application of anti–PD-1/PD-L1 treatment in thyroid cancer (6–8, 57). Interestingly, the ORR to immunotherapy was nearly 10% and 20% in PTC and ATC, respectively, while the biological reason for this difference was unexplored. Previous studies highlighted classic immunotherapy markers, including tumor mutation burden, PD-L1 expression, microsatellite instability, and so on (58). However, in the era of scRNA-Seq, key cell subpopulations in the TME were identified to be essential for the immunotherapy response, and CXCL13^+^ T cells emerged as one of the most important T lymphocyte subgroups in cancer immunotherapy. For example, CXCL13^+^ T cells were reported to predict an effective PD-L1 blockade response in triple-negative breast cancer (59). Moreover, a meta-analysis also demonstrated that CXCL13^+^ T cells could be tumor-reactive T cells to ICIs in the treatment of multiple solid tumors (60). Based on these findings, we uncovered the enrichment of CXCL13^+^ exhausted T cells in ATC, suggesting their central role in the response of ATC to immunotherapy.

Previous studies showed that CXCL13^+^ T cells could advance the development of early TLSs in cancers (61). A TLS refers to the ectopic lymphoid organ in nonlymphoid tissues during chronic inflammation and cancer development, which is believed to be correlated with prognosis and sensitivity to ICIs in various cancers (62, 63). For example, the presence of 2 or more TLSs predicted better progression-free survival in a cohort of clear cell renal cancer patients receiving nivolumab (64). Nevertheless, detailed information on the contribution of TLSs to improving immunotherapy is still unclear. It was reported that immunologic memory and activation of cytotoxic T cells could be fostered in TLSs, and local B cells might also produce tumor-reactive IgGs (65, 66).

Of note, TLS formation in the thyroid gland has been formerly characterized in HT, which is a chronic autoimmune disease (60, 67). However, the potential role of TLSs in thyroid cancer has rarely been reported. In this study, we directly identified lymphocyte aggregates and early TLSs in ATC patients by mIHC. Besides, we also revealed a different biological process of developing TLSs in HT. These findings indicated that the microenvironment of ATC facilitated TLS development in thyroid cancer instead of HT. The early TLS development could be explained by the activated lymphotoxin pathway and enrichment of cell adhesion molecules in ATC (68). To our knowledge, this is the first study to demonstrate immature TLSs in ATC and the role of CXCL13^+^ T cells in ATC development, which offers a potential explanation for the sensitivity of ATC to anti–PD-1/PD-L1 treatment. In contrast, for autoimmune thyroiditis, we further highlighted the chemokine-positive stromal cells’ essential contribution to advance TLS in HT, which is also consistent with previous reports (26). Thyroid cancer patients with HT might be more sensitive to immunotherapy, since more TLSs could be fostered in autoimmune thyroiditis. Still, clinical cohort studies should be conducted to validate the hypothesis.

Although ICIs performed better in ATC than in PTC, the overall ORR to anti–PD-1/PD-L1 antibodies was still less than 30% (6, 7). As was reported in clinical trials, the ORR of ATC was lower than 20%. Although we found potential immature TLSs in ATC, the ATC is still a group of highly heterogeneous diseases. The level of conventional immunotherapy biomarkers (like PD-L1 and TMB) was discrepant among individual ATC cases (69, 70). Also, the infiltration pattern of CXCL13^+^ T cells varied among ATC samples included in the study, which indicated the different possibilities for early TLS development. Similarly, several ATC cases also had a lower score of classic TLS signatures, based on public microarray data analysis. Besides, the immaturity of TLSs might also hamper immunotherapy response, since we mainly identified early TLSs in ATC. Mature TLSs were reported to better foster anticancer immunity (32). Therefore, the induction and maturation of TLSs could be a promising way to improve cancer immunotherapy (71), and we aimed to explore some potential solution to promote TLS development in thyroid cancer. We observed severe tumor vessel abnormalities in ATC, but normal veins in the tumor were essential for TLS development (39). Moreover, HEVs, which could recruit key immune cells into TLSs (40), were rarely present around the immature TLSs in ATC. Thus, vascular normalization and HEV induction could be orchestrated in thyroid cancer for TLS formation to improve the effect of immunotherapy.

Famitinib, an antiangiogenic agent and a novel anticancer agent, covers multiple targets, including VEGFR-2 and -3, PDGFR-β, c-kit, FLT3, RET, and TAM family of kinases (AXL and MER) (72). It was previously reported to cause subclinical/clinical hypothyroidism in patients with cancer, which might be induced by thyroiditis (73). The mechanism of induced thyroiditis remains unclear, but reports have shown that antiangiogenic agents can remodel thyroid vasculatures and immune infiltration (73, 74). Of note, the incidence of hypothyroidism triggered by famitinib plus camrelizumab was rather high compared with other antiangiogenic agents (75, 76). This indicated that famitinib could normalize the vessels in thyroid tumors, and famitinib plus ICIs might promote TLS development to improve immunotherapy. Then, we performed an in vivo experiment using an immune-competent murine thyroid cancer model. We found that the combined therapy could potentiate TLS development by improving B cell infiltration and upregulating TLS molecular inducers in BPC allografts. In addition, we also bridged famitinib plus camrelizumab in clinical use. After combination therapy, the CXCL13^+^ T cells in a patient with ATC had memory-like features, which were similar to those of tumor-reactive T lymphocytes from immunotherapy responders in other cancers (60, 77). Furthermore, we verified that the combined therapeutic strategy induced mature TLS development and significantly restricted tumor growth in patients with ATC through a clinical trial (Figure 8). These results provided us insights into the synergistic effect that famitinib and immunotherapy might have on thyroid cancer.

In summary, we profiled in detail the TME of PTC and ATC via scRNA-Seq. Various cell lineages and their contribution to the aggressiveness of ATC were also displayed in this research. Moreover, we first identified the key CXCL13^+^ T cells and early TLSs in ATC that could make ATC more sensitive to immunotherapy, implying that promoting TLS development could be a promising way to improve the efficacy of immunotherapy for thyroid cancer.

## Methods

### Sex as a biological variable.

Both female and male animals were used in the study, but sex was not considered as a biological variable.

### Sample collection and scRNA-Seq processing.

The scRNA-Seq data of PTC primary tumors and metastases were obtained from our previous scientific project (27). In addition, we downloaded another scRNA-Seq ATC and PTC data set from the National Center for Biotechnology Gene Expression Omnibus (GEO) database (GSE193581) (78). Additional treatment-naive ATC primary tumor samples were obtained from 3 patients with ATC treated in our department (A34, A35, and A36). Another sample from a patient with ATC who received camrelizumab and famitinib was collected after her surgery (A37). The clinical features of these patients are displayed in Supplemental Table 1. In addition, another independent scRNA-Seq data set of 4 HT patients was collected from Genome Sequence Archive (HRA001684) (26).

Before conducting scRNA-Seq, biopsy samples were prepared according to our previous research (27). Next, single-cell libraries of dissociated cell suspensions were established with Chromium Single Cell 3’ Reagent Kits (10x Genomics). The single-cell libraries were then sequenced on an Illumina NovaSeq 6000 System. The raw sequencing data were aligned to the GRCh38 human genome, and gene barcode count matrices were generated by Cell Ranger (v5.0.0).

### Single-cell data quality control and integrated analysis.

Overall, we collected data from 48 samples from 21 patients with PTC and 13 patients with ATC. First, we filtered the scRNA-Seq data of each sample with the R package Seurat (v4.0.2) according to the following criteria: percentage of mitochondrial gene counts < 10%, unique molecular identifiers > 300, and genes > 200. We applied the R package DoubletFinder (v2.0) to filter out and remove the doublets.

The transcriptomic profiles of all untreated samples were integrated with the “merge” function. Next, the “NormalizeData” function was employed to normalize the sequencing data. A total of 2,000 highly variable genes were identified with the “FindVariableFeatures” function. Sequencing data were then scaled to these highly variable genes. Principal component analysis was conducted, and we selected the top 20 principal components for later analysis, according to the elbow plot generated by the “ElbowPlot” function. To correct the batch effect, we utilized the “Runharmony” function from the harmony package (79). Subsequently, we used “FindNeighbors” and “FindClusters” to cluster cells in an unsupervised manner. The marker genes of cell clusters were found by the “FindAllMarkers” function from Seurat and the “cosg” function from the COSG package. Then, we first identified 6 major cell lineages. Next, we conducted a similar analysis procedure to further detect subgroups of tumor cells, immune cells, and stromal cells. For the scRNA-Seq data of A37 and patients with HT, a similar analysis procedure was conducted separately.

### Determination of DEGs and pathway enrichment analysis.

We used the MAST method to identify the DEGs of different cell clusters (80). DEGs were determined as follows: |logFC| > 0.6 and differential percentage > 0. The “EnhancedVolcano” function was applied to draw volcano plots to show the key DEGs found above. Next, we performed preranked GSEA or GSVA with the R packages clusterProfiler and GSVA (81).

Gene set scores of single-cell data were calculated by the “AddModuleScore” function from the Seurat package. For bulk RNA-Seq data, we applied MCP-counter to evaluate the scores of several gene models.

### Construction of the ATC-Epi gene signature.

The DEGs of ATC-Epis compared with PTC-Epis were obtained by the function mentioned above. Next, we selected the top regulated genes, following the criteria logFC > 1.5, differential percentage > 0.15, and *P* value < 0.01, to construct the ATC-Epi–specific signature. To validate the high expression of this signature in ATC, we collected 5 other independent microarray data sets of PTC and ATC from the GEO database (GSE29265, GSE33630, GSE53157, GSE65144, and GSE76030) (69, 82–84). Moreover, we tested the negative prognostic value of this ATC-Epi–specific signature in TCGA-THCA database with a web-based tool named GEPIA2 (http://gepia2.cancer-pku.cn/).

### Identification of regulatory TFs.

The top regulons of cell lineages were determined by the DoRothEA R package (v1.3.3) (85). TFs with A to D confidence levels were used for subsequent analysis. Next, the “run_viper” function evaluated TF activity with default settings.

### Cell-cell interaction analysis.

To explore dense cell-cell interaction niches in ATC and PTC, we used CellChat to develop a cell-cell communication analysis (86). The human L-R pair repertoire was also obtained from the CellChat package. The *P* value and communication probability were calculated by the standard procedure (https://github.com/sqjin/CellChat/blob/master/tutorial/Comparison_analysis_of_multiple_datasets_with_different_cellular_compositions.html; commit ID e2acde7). Finally, we applied the “netVisual_bubble” function to visualize vital cell-cell interactions that differed significantly between ATC and PTC samples. In addition, the cell-cell interaction analysis of A37 and HT data sets was performed solely with CellChat.

### Trajectory analysis.

We applied the monocle2 (v2.9.0) or monocle3 package (v1.3.1) to conduct pseudotime analysis. High-dispersion genes were regarded as ordering genes in the later monocle2 analysis, following this criterion: dispersion_empirical > 2 × dispersion_fit. The root state was determined by the high expression of naive T cell markers or calculated by the “get_earliest_principal_node” function from monocle3. The exhaustion score was calculated by the “AddModuleScore” function from Seurat.

### HE staining and IHC.

Formalin-fixed, paraffin-embedded sections from patients with ATC were provided by the Department of Pathology at Fudan University Shanghai Cancer Centre (FUSCC). HE staining was also performed by pathologists from the Department of Pathology.

Following the manufacturer’s protocol, we performed mIHC with a tyramide signal amplification (TSA) kit (abs50029, Absin). All sections were dewaxed in xylene and then rehydrated in an ethanol series. Antigen retrieval was conducted in citrate sodium buffer in a steam heater for 15 minutes. Then, sections were incubated with 3% H_2_O_2_ for 10 minutes to remove endogenous peroxidases. A 3% BSA (Beyotime) solution was used for blocking. The primary antibody was then diluted in PBS, and sections were then incubated with the antibody solution at 4°C overnight. After primary antibody incubation, the secondary goat anti-rabbit antibody from the TSA kit was used. Finally, a fluorescent dye was used to stain horseradish peroxidase (HRP). Binding antibodies were first removed by conducting antigen retrieval, and we repeated the procedure 4 times. Finally, a DAPI solution was used to stain the nucleus. The primary antibodies used in mIHC included anti-CD4 (1:100, 48274S, Cell Signaling Technology), anti-CD8A (1:200, 85336S, Cell Signaling Technology), anti-CD20 (1:200, 48750S, Cell Signaling Technology), and anti-CXCL13 (1:1,000, ab246518, Abcam) antibodies.

Fresh mouse tumor specimens were fixed in 4% paraformaldehyde for 24–48 hours and then embedded and sliced into 4 μM sections. For conventional IHC, sections were first dewaxed and rehydrated. Antigen retrieval and blocking processes were the same as for mIHC. Then, sections were incubated with diluted primary antibodies at 4°C overnight. HRP-linked secondary antibody was then incubated for 30 minutes at room temperature. Afterward, we applied a diaminobenzidine solution for staining HRP. The primary antibodies used in IHC included anti-MECA79 (1:50, sc-19602, Santa Cruz Biotechnology), anti-Cxcl13 (1:1,000, ab199043, Abcam), and anti-TTF1 (1:500, ab76012, Abcam) antibodies. The anti-rabbit/mouse HRP-linked secondary antibody and diaminobenzidine solution were obtained from a ready-to-use IHC kit (GK500075, Genentech, Roche). The anti-rat HRP-linked antibody (1:500, abs20031) was purchased from Absin. IHC images were obtained with an Olympus SLIDEVIEW VS200 digital slide scanner and viewed by Olympus Olyvia (v3.3).

### Murine primary ATC cell line construction.

In vivo experiments were conducted using immune-competent models. As no commercially available murine thyroid cancer cell lines have been available until now, we constructed *Braf^CA/+^ Trp53^fl/fl^ Tpo-CreER^T2^* transgenic mice to develop a primary thyroid cancer cell line (BPC). Tg (TPO-cre/ERT2)1139Tyj (strain 026512) and B6;129-Braf tm1Mmcm/+ (strain 017837) mice were obtained from The Jackson Laboratory. Conditional Trp53-knockout model mice were described previously (87, 88).

The BPC cell line was derived from the thyroid of a BPC mouse. Primary thyroid cells were separated and cultured as previously described (89). The first 3 passages were cultured in F-12 medium with 5 μg/mL transferrin (40133ES60, Yeasen Biotech), 10 μg/mL bovine insulin (BS001, Biosharp), 3.5 ng/mL hydrocortisone (G8450, Solarbio), 2 ng/mL glycyl-l-histidyl-l-lysine (HY-P0046, MedChemExpress), and 10 ng/mL somatostatin (MB1225, Meilunbio).

### Animal ultrasound imaging.

Fujifilm Vevo 2100 was adopted to monitor the thyroid morphology of mice. The MS400 detector was used to scan the neck region.

### Cell culture and construction of immunocompetent mouse xenografts.

The MC38 cell line was obtained from ATCC. Cell lines were cultured in DMEM (Gibco, Invitrogen) with 10% FBS (Gibco, Invitrogen) and grown at 37°C in a humidified atmosphere containing 5% CO_2_. C57BL/6 wild-type mice 4–6 weeks old were obtained from the Laboratory Animal Center of FUSCC and housed under specific pathogen–free conditions.

The mice were inoculated subcutaneously in the flank with 4 × 10^6^ MC38 or BPC tumor cells in DMEM mixed with Matrigel (Corning). Tumor volume was calculated using the following formula: volume (mm^3^) = (length) × (width) × (width)/2. When tumor volumes reached 60–100 mm^3^, mice were randomly divided into different groups. We measured the tumor volume of each mouse every 2–4 days. Mice were sacrificed, and tumors were removed when the largest tumor reached a volume of 1,000 mm^3^.

### Drug administration.

For mouse experiments, famitinib, a gift from Jiangsu Hengrui Pharmaceuticals Co., Ltd (Lianyungang, China), was orally (p.o.) administered at a dose of 30 mg/kg each day (90). Mice were intraperitoneally injected with 200 μg anti-mouse PD-1 antibody (BE0146, BioXCell) or isotype antibody (BE0089, BioXCell) twice a week (91).

For clinical trials, enrolled patients with thyroid cancer received camrelizumab 200 mg intravenously on day 1 of each 21-day cycle and famitinib 20 mg p.o. once a day in 21-day cycles until the cancer progressed or the patient could not tolerate the drug (NCT04521348).

### Flow cytometry analysis.

Removed tumors from murine models were cut into pieces and digested in PBS buffer with DNase I (Roche) and collagenase IV (MilliporeSigma) for 1 hour at 37°C. Subsequently, tumor pieces were passed through a 70 μm strainer (Falcon, Corning) to prepare a single-cell suspension. Subsequently, the cell surface was stained in FACS buffer (FBS) with fluorescently labeled antibodies, including anti-CD45 (103115, BioLegend), anti-CD4 (100433, BioLegend), anti-CD8A (100721, BioLegend), anti–PD-1 (135227, BioLegend), and anti-CD19 (11-0193-82, Invitrogen, Thermo Fisher Scientific) antibodies. Flow cytometry was conducted on a Cytoflex S cell analyzer (Beckman Coulter). Data analysis was carried out using CytExpert software (v2.3).

### QPCR.

We first extracted total RNA from fresh tumor xenograft tissue with a TRIzol kit (0581S, CWBio). Then, cDNA was generated by reverse transcriptase (2020S, CWBio). QPCR was performed with Magic-SYBR Mixture (3008S, CWBio) on the CFX96 touch system (Bio-Rad). We used β-actin as the internal reference for quantification. Primers for *Cxcl13*, *Icam1*, *Vcam1*, *Ltb*, and *Ltbr* were purchased from Sangon Biotech. Co. Ltd. (Shanghai, China). Sequences of primers are shown in Supplemental Table 7.

### Western blot.

Murine tumor specimens were lysed in RIPA buffer mixed with protease and phosphatase inhibitors (78443, Invitrogen, Thermo Fisher Scientific) and then crushed by an ultrasonic pulverizer (Sonics). After centrifugation (15 minutes, at 16,000*g*, at 4°C) and denaturation, the total proteins of tumor xenografts were extracted. After quantification by a BCA protein assay kit (P0012, Beyotime), 6–8 μL protein extract was separated by 4%–20% SDS-polyacrylamide gel (M00656, GenScript) electrophoresis. Subsequently, proteins were transferred to polyvinylidene fluoride (PVDF) membranes (IPVH00010, MilliporeSigma). After blocking in nonfat milk, PVDF membranes were incubated with primary antibodies at 4°C overnight. Primary antibodies used in Western blotting included anti-MECA79 (1:100, sc-19602, Santa Cruz Biotechnology), anti-Cxcl13 (1:1,000, bs-4509R, Bioss), and anti–β-actin (1:1,000, 4970S, Cell Signaling Technology) antibodies. Subsequently, anti-rat (1:1,000, abs20031, Absin) or anti-rabbit (1:1,000, ab205718, Abcam) HRP-conjugated secondary antibodies were applied to the PVDF membranes. The protein bands were visualized by Immobilon Western HRP Substrate (MilliporeSigma) and detected through a fluorescence chemiluminescence gel imaging system. The results of the Western blot were quantified by ImageJ (v1.53q, NIH).

### Statistics.

All the statistical analyses included in the study were performed with R (v4.0.3) or GraphPad Prism (v8.0.2). Data are presented as the mean ± standard deviation of at least 3 independent replications. Two-tailed Student’s *t* test or the Mann-Whitney *U* test was used for the comparison of 2 groups. The measurement data in multiple groups were compared with 1-way ANOVA or the Kruskal-Wallis test. The *P* value of pairwise comparisons was adjusted by an adaptive FDR procedure or Tukey’s multiple-comparison test (92). A *P* value less than 0.05 was regarded as significant.

### Study approval.

The Institutional Review Board of Fudan University Shanghai Cancer Center approved this study, which was performed in accordance with the Declaration of Helsinki. Every enrolled patient signed an informed consent document before sample collection. The protocol of animal experiments was approved by the Institutional Animal Care and Use Committee of Fudan University Shanghai Cancer Center.

### Data availability.

The scRNA-Seq data supporting this study have been deposited in CNGB Nucleotide Sequence Archive of China National GeneBank (https://db.cngb.org/cnsa/). The accession number is CNP0004262. Publicly available scRNA-Seq data sets of thyroid cancer were downloaded from GEO (GSE184362, GSE193581). Microarray data of thyroid cancer were also obtained from GEO (GSE29265, GSE33630, GSE53157, GSE65144, GSE76030). The scRNA-Seq data set of 4 patients with HT was collected from Genome Sequence Archive in National Genomics Data Center, China National Center for Bioinformation/Beijing Institute of Genomics, Chinese Academy of Sciences (HRA001684). Values for all data points found in graphs are in the Supporting Data Values file.

## Author contributions

PZH and YLW designed the entire project. PZH, XS, PCY, and YLW wrote and reviewed the manuscript. PZH, WDY, PCY, LCT, XFC, and CH performed the experiments. PZH, PCY, and LCT analyzed and interpreted the data. JQH, WJW, ZWL, NQ, YW, QHJ, and YLW supervised the study. DMJ and YLW provided key resources for this study. The authorship order among co–first authors is as follows: PZH (first), WDY (second), PCY (third), LCT (fourth), and XS (fifth) based on their contributions to drafting the manuscript. All the authors have read and approved the manuscript.

## Supplementary Material

Supplemental data

Unedited blot and gel images

Supplemental tables 1-7

Supporting data values

## Version 1 03/13/2024

In-Press Preview

## Version 2 04/22/2024

Electronic publication

## References

[1] Sung H (2021). Global cancer statistics 2020: GLOBOCAN estimates of incidence and mortality worldwide for 36 cancers in 185 countries. CA Cancer J Clin.

[2] Miranda-Filho A (2021). Thyroid cancer incidence trends by histology in 25 countries: a population-based study. Lancet Diabetes Endocrinol.

[3] Maniakas A (2020). Evaluation of overall survival in patients with anaplastic thyroid carcinoma, 2000-2019. JAMA Oncol.

[4] Saini S (2018). Therapeutic advances in anaplastic thyroid cancer: a current perspective. Mol Cancer.

[5] Ohaegbulam KC (2015). Human cancer immunotherapy with antibodies to the PD-1 and PD-L1 pathway. Trends Mol Med.

[6] Capdevila J (2020). PD-1 blockade in anaplastic thyroid carcinoma. J Clin Oncol.

[7] Mehnert JM (2019). Safety and antitumor activity of the anti-PD-1 antibody pembrolizumab in patients with advanced, PD-L1-positive papillary or follicular thyroid cancer. BMC Cancer.

[8] Tabernero J (2022). Phase II multicohort study of atezolizumab monotherapy in multiple advanced solid cancers. ESMO Open.

[9] Garcia-Alvarez A (2022). What is the status of immunotherapy in thyroid neoplasms?. Front Endocrinol (Lausanne).

[10] Lei Y (2021). Applications of single-cell sequencing in cancer research: progress and perspectives. J Hematol Oncol.

[11] Zhang M (2021). Dissecting transcriptional heterogeneity in primary gastric adenocarcinoma by single cell RNA sequencing. Gut.

[12] Li J (2022). Single-cell characterization of the cellular landscape of acral melanoma identifies novel targets for immunotherapy. Clin Cancer Res.

[13] Missero C (1998). Molecular events involved in differentiation of thyroid follicular cells. Mol Cell Endocrinol.

[14] Kapeleris J (2022). Modelling reoxygenation effects in non-small cell lung cancer cell lines and showing epithelial-mesenchymal transition. J Cancer Res Clin Oncol.

[15] Schuster A (2020). AN1-type zinc finger protein 3 (ZFAND3) is a transcriptional regulator that drives Glioblastoma invasion. Nat Commun.

[16] Weinberger P (2017). Cell cycle M-phase genes are highly upregulated in anaplastic thyroid carcinoma. Thyroid.

[17] López-Márquez A (2022). Sox9 is involved in the thyroid differentiation program and is regulated by crosstalk between TSH, TGFβ and thyroid transcription factors. Sci Rep.

[18] Ma R (2020). A stem cell surge during thyroid regeneration. Front Endocrinol (Lausanne).

[19] Hu H (2017). Acetylation of PGK1 promotes liver cancer cell proliferation and tumorigenesis. Hepatology.

[20] Wen B (2020). LOXL2 in cancer: regulation, downstream effectors and novel roles. Biochim Biophys Acta Rev Cancer.

[21] Goveia J (2020). An integrated gene expression landscape profiling approach to identify lung tumor endothelial cell heterogeneity and angiogenic candidates. Cancer Cell.

[22] Rohlenova K (2018). Endothelial cell metabolism in health and disease. Trends Cell Biol.

[23] Perez VM (2021). The PDAC extracellular matrix: a review of the ECM protein composition, tumor cell interaction, and therapeutic strategies. Front Oncol.

[24] Lin CL (2021). Platelet-derived growth factor receptor-α subunit targeting suppresses metastasis in advanced thyroid cancer in vitro and in vivo. Biomol Ther (Seoul).

[25] Ben-Skowronek I (2011). Interactions of lymphocytes, thyrocytes and fibroblasts in Hashimoto’s thyroiditis: an immunohistochemical and ultrastructural study. Horm Res Paediatr.

[26] Zhang QY (2022). Lymphocyte infiltration and thyrocyte destruction are driven by stromal and immune cell components in Hashimoto’s thyroiditis. Nat Commun.

[27] Bassez A (2021). A single-cell map of intratumoral changes during anti-PD1 treatment of patients with breast cancer. Nat Med.

[28] Si J (2020). Hematopoietic progenitor kinase1 (HPK1) mediates T cell dysfunction and is a druggable target for T cell-based immunotherapies. Cancer Cell.

[29] Guo X (2018). Global characterization of T cells in non-small-cell lung cancer by single-cell sequencing. Nat Med.

[30] Mittelbrunn M, Kroemer G (2021). Hallmarks of T cell aging. Nat Immunol.

[31] Figenschau SL (2018). ICAM1 expression is induced by proinflammatory cytokines and associated with TLS formation in aggressive breast cancer subtypes. Sci Rep.

[32] Helmink BA (2020). B cells and tertiary lymphoid structures promote immunotherapy response. Nature.

[33] Coppola D (2011). Unique ectopic lymph node-like structures present in human primary colorectal carcinoma are identified by immune gene array profiling. Am J Pathol.

[34] Hennequin A (2016). Tumor infiltration by Tbet+ effector T cells and CD20+ B cells is associated with survival in gastric cancer patients. Oncoimmunology.

[35] Cillo AR (2020). Immune landscape of viral- and carcinogen-driven head and neck cancer. Immunity.

[36] Giannini R (2019). Immune profiling of thyroid carcinomas suggests the existence of two major phenotypes: an ATC-like and a PDTC-like. J Clin Endocrinol Metab.

[37] Meylan M (2020). Early hepatic lesions display immature tertiary lymphoid structures and show elevated expression of immune inhibitory and immunosuppressive molecules. Clin Cancer Res.

[38] Rydzewska M (2018). Role of the T and B lymphocytes in pathogenesis of autoimmune thyroid diseases. Thyroid Res.

[39] Hiraoka N (2015). Intratumoral tertiary lymphoid organ is a favourable prognosticator in patients with pancreatic cancer. Br J Cancer.

[40] Vella G (2021). High endothelial venules: a vascular perspective on tertiary lymphoid structures in cancer. Front Immunol.

[41] McFadden DG (2014). p53 constrains progression to anaplastic thyroid carcinoma in a Braf-mutant mouse model of papillary thyroid cancer. Proc Natl Acad Sci U S A.

[42] Sautès-Fridman C (2019). Tertiary lymphoid structures in the era of cancer immunotherapy. Nat Rev Cancer.

[43] Kallies A (2008). Distinct regulation of effector and memory T-cell differentiation. Immunol Cell Biol.

[44] Peeters BWA, Gillespie GM (2023). Adaptive meets innate: CD8^+^ T cells kill MHC-I-negative tumour cells. Nat Rev Immunol.

[45] Ranganath R (2015). Anaplastic thyroid cancer. Curr Opin Endocrinol Diabetes Obes.

[46] Romitti M (2013). Signaling pathways in follicular cell-derived thyroid carcinomas (review). Int J Oncol.

[47] Talbott I (2015). Undifferentiated (anaplastic) thyroid carcinoma: practical immunohistochemistry and cytologic look-alikes. Semin Diagn Pathol.

[48] Yu P (2023). TERT accelerates BRAF mutant-induced thyroid cancer dedifferentiation and progression by regulating ribosome biogenesis. Sci Adv.

[49] Affo S (2021). Promotion of cholangiocarcinoma growth by diverse cancer-associated fibroblast subpopulations. Cancer Cell.

[50] Li X (2022). Single-cell RNA sequencing reveals a pro-invasive cancer-associated fibroblast subgroup associated with poor clinical outcomes in patients with gastric cancer. Theranostics.

[51] Gajewski TF (2013). Innate and adaptive immune cells in the tumor microenvironment. Nat Immunol.

[52] Jiang X (2019). Role of the tumor microenvironment in PD-L1/PD-1-mediated tumor immune escape. Mol Cancer.

[53] Mantovani A (2002). Macrophage polarization: tumor-associated macrophages as a paradigm for polarized M2 mononuclear phagocytes. Trends Immunol.

[54] Maier B (2020). A conserved dendritic-cell regulatory program limits antitumour immunity. Nature.

[55] La-Beck NM (2015). Immune checkpoint inhibitors: new insights and current place in cancer therapy. Pharmacotherapy.

[56] Barber DL (2006). Restoring function in exhausted CD8 T cells during chronic viral infection. Nature.

[57] Marabelle A (2020). Association of tumour mutational burden with outcomes in patients with advanced solid tumours treated with pembrolizumab: prospective biomarker analysis of the multicohort, open-label, phase 2 KEYNOTE-158 study. Lancet Oncol.

[58] Morand S (2021). Ovarian cancer immunotherapy and personalized medicine. Int J Mol Sci.

[59] Zhang Y (2021). Single-cell analyses reveal key immune cell subsets associated with response to PD-L1 blockade in triple-negative breast cancer. Cancer Cell.

[60] Liu B (2022). Single-cell meta-analyses reveal responses of tumor-reactive CXCL13^+^ T cells to immune-checkpoint blockade. Nat Cancer.

[61] Ukita M (2022). CXCL13-producing CD4^+^ T cells accumulate in the early phase of tertiary lymphoid structures in ovarian cancer. JCI Insight.

[62] Fu T (2021). Spatial architecture of the immune microenvironment orchestrates tumor immunity and therapeutic response. J Hematol Oncol.

[63] Li H (2023). Tertiary lymphoid structures and cytokines interconnections: The implication in cancer immunotherapy. Cancer Lett.

[64] Carril-Ajuria L (2022). Baseline circulating unswitched memory B cells and B-cell related soluble factors are associated with overall survival in patients with clear cell renal cell carcinoma treated with nivolumab within the NIVOREN GETUG-AFU 26 study. J Immunother Cancer.

[65] Meylan M (2022). Tertiary lymphoid structures generate and propagate anti-tumor antibody-producing plasma cells in renal cell cancer. Immunity.

[66] Noël G (2021). Functional Th1-oriented T follicular helper cells that infiltrate human breast cancer promote effective adaptive immunity. J Clin Invest.

[67] Marinkovic T (2006). Interaction of mature CD3^+^CD4^+^ T cells with dendritic cells triggers the development of tertiary lymphoid structures in the thyroid. J Clin Invest.

[68] Tang H (2017). Lymphotoxin signalling in tertiary lymphoid structures and immunotherapy. Cell Mol Immunol.

[69] Landa I (2016). Genomic and transcriptomic hallmarks of poorly differentiated and anaplastic thyroid cancers. J Clin Invest.

[70] Chowdhury S (2016). Programmed death-ligand 1 overexpression is a prognostic marker for aggressive papillary thyroid cancer and its variants. Oncotarget.

[71] Johansson-Percival A (2017). De novo induction of intratumoral lymphoid structures and vessel normalization enhances immunotherapy in resistant tumors. Nat Immunol.

[72] Qu YY (2021). Camrelizumab plus famitinib in patients with advanced or metastatic renal cell carcinoma: data from an open-label, multicenter phase II basket study. Clin Cancer Res.

[73] Cao J (2014). Hypothyroidism as a potential biomarker of efficacy of famitinib, a novel VEGFR-2 inhibitor in metastatic breast cancer. Cancer Chemother Pharmacol.

[74] Alexandrescu DT (2008). Sunitinib-associated lymphocytic thyroiditis without circulating antithyroid antibodies. Thyroid.

[75] Chen L (2022). Famitinib with camrelizumab and nab-paclitaxel for advanced immunomodulatory triple-negative breast cancer (FUTURE-C-plus): an open-label, single-arm, phase II trial. Clin Cancer Res.

[76] De Leo S (2023). Endocrine-related adverse conditions induced by tyrosine kinase inhibitors. Ann Endocrinol (Paris).

[77] Huang Q (2022). The primordial differentiation of tumor-specific memory CD8^+^ T cells as bona fide responders to PD-1/PD-L1 blockade in draining lymph nodes. Cell.

[78] Lu L (2023). Anaplastic transformation in thyroid cancer revealed by single-cell transcriptomics. J Clin Invest.

[79] Korsunsky I (2019). Fast, sensitive and accurate integration of single-cell data with Harmony. Nat Methods.

[80] Finak G (2015). MAST: a flexible statistical framework for assessing transcriptional changes and characterizing heterogeneity in single-cell RNA sequencing data. Genome Biol.

[81] Yu G (2012). clusterProfiler: an R package for comparing biological themes among gene clusters. OMICS.

[82] Tomás G (2012). A general method to derive robust organ-specific gene expression-based differentiation indices: application to thyroid cancer diagnostic. Oncogene.

[83] Pita JM (2009). Gene expression profiling associated with the progression to poorly differentiated thyroid carcinomas. Br J Cancer.

[84] Von Roemeling CA (2015). Aberrant lipid metabolism in anaplastic thyroid carcinoma reveals stearoyl CoA desaturase 1 as a novel therapeutic target. J Clin Endocrinol Metab.

[85] Holland CH (2020). Robustness and applicability of transcription factor and pathway analysis tools on single-cell RNA-seq data. Genome Biol.

[86] Jin S (2021). Inference and analysis of cell-cell communication using CellChat. Nat Commun.

[87] Han X (2014). Transdifferentiation of lung adenocarcinoma in mice with Lkb1 deficiency to squamous cell carcinoma. Nat Commun.

[88] Jacks T (1994). Tumor spectrum analysis in p53-mutant mice. Curr Biol.

[89] Jeker LT (1999). Mouse thyroid primary culture. Biochem Biophys Res Commun.

[90] Ge S (2016). Famitinib exerted powerful antitumor activity in human gastric cancer cells and xenografts. Oncol Lett.

[91] Li N (2022). ARID1A loss induces polymorphonuclear myeloid-derived suppressor cell chemotaxis and promotes prostate cancer progression. Nat Commun.

[92] Benjamini Y (2006). Adaptive linear step-up procedures that control the false discovery rate. Biometrika.

