# Supplementary material for: A distinct tumor microenvironment makes anaplastic thyroid cancer more lethal but immunotherapy sensitive than papillary thyroid cancer
Source: JCI Insight. 2024 Mar 13;9(8):e173712. doi: 10.1172/jci.insight.173712 (PMC11141884; doi:10.1172/jci.insight.173712)

Supplemental Figures

Supplemental Figure 1. The cellular landscape of PTC and ATC.

A. UMAP plot of qualified 226066 cells colored by sample names.

B. Normalized expression of top 50 upregulated genes for each major cell lineage.

C. Bar plot depicted the proportion of each major cell lineage in 48 samples.

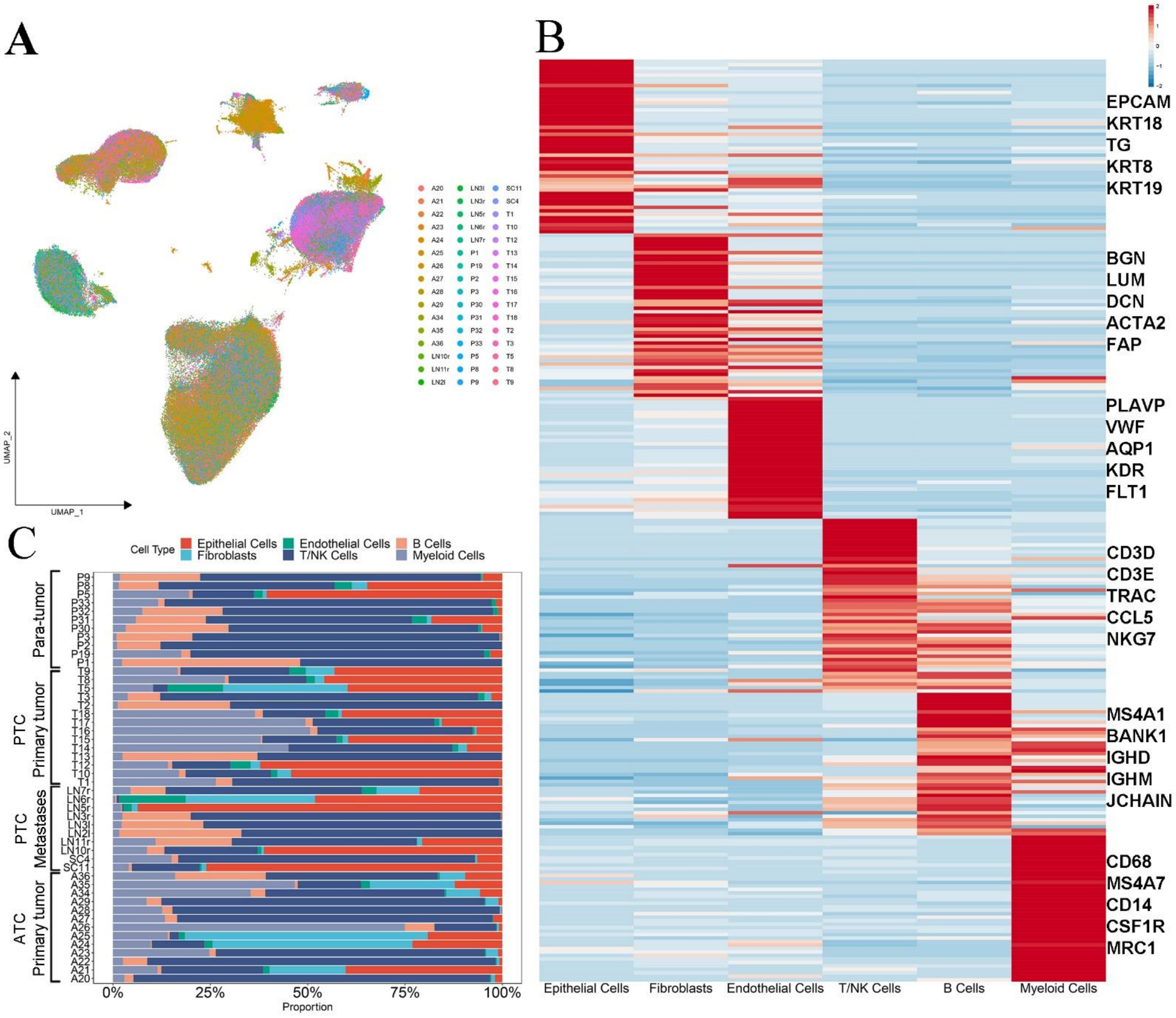

Supplemental Figure 2. Two major subgroups of epithelial cells from PTC and ATC primary tumor samples.

A. Unsupervised clustering analysis revealed 15 clusters of epithelial cells.

B. UMAP plot of identified epithelial cells colored by sample names.

C. Feature plots showed maker genes for PTC-Epi and ATC-Epi.

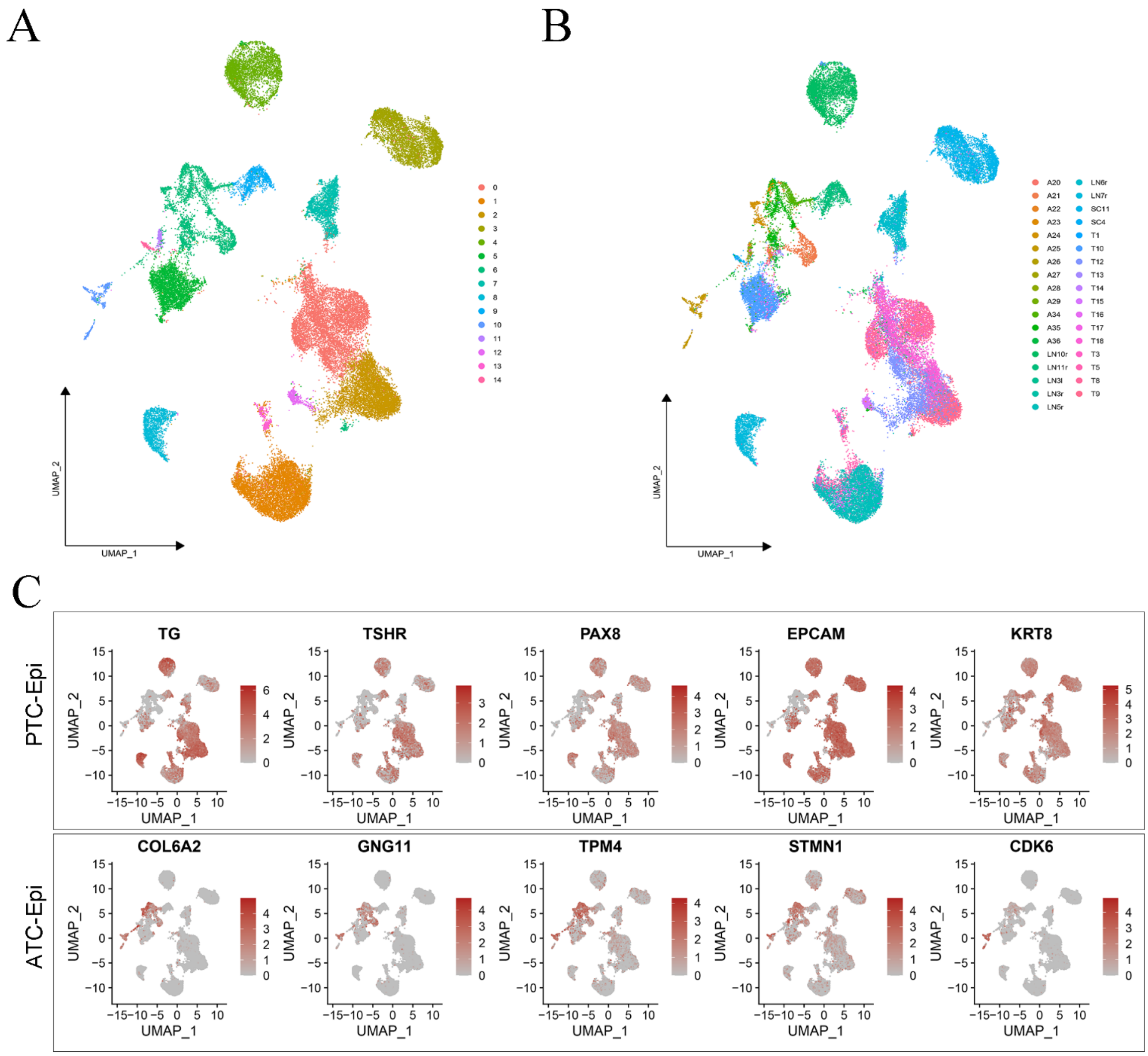

Supplemental Figure 3. TMSB4X, a potential shared biomarker for ATC-Epis and PTC-Epis.

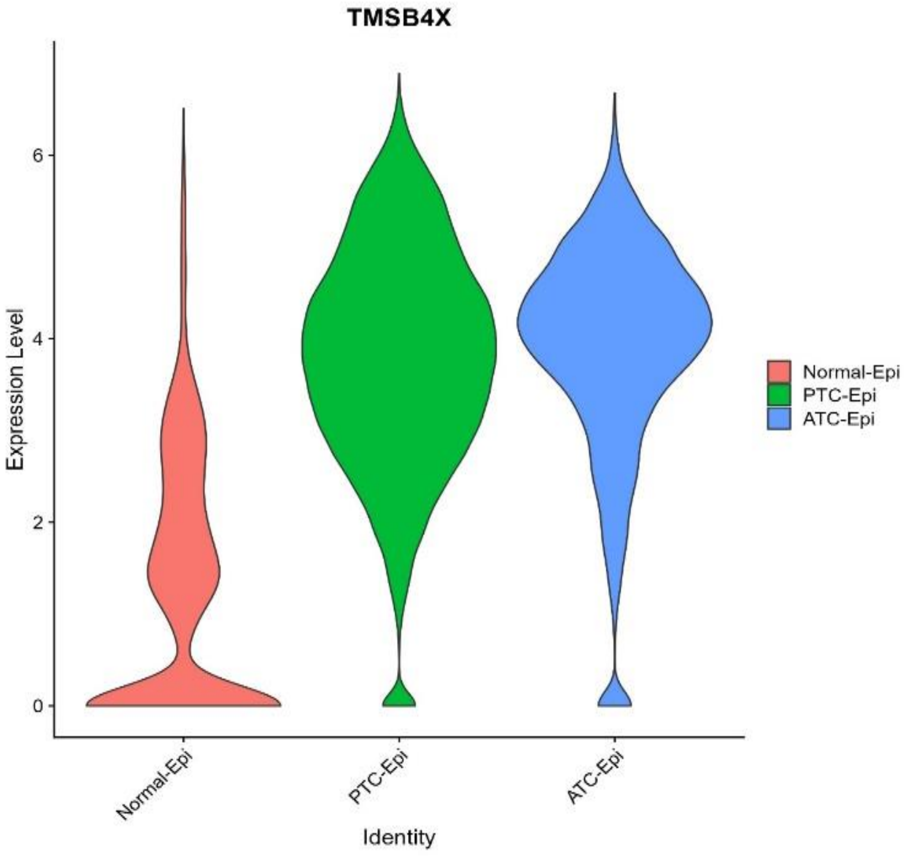

Supplemental Figure 4. Trajectory analysis of the epithelial lineage.

A. UMAP plot of epithelial cells colored by pseudotime with potential trajectory.

B. Negative correlation between the level of thyroid differentiated markers expression and pseudotime.

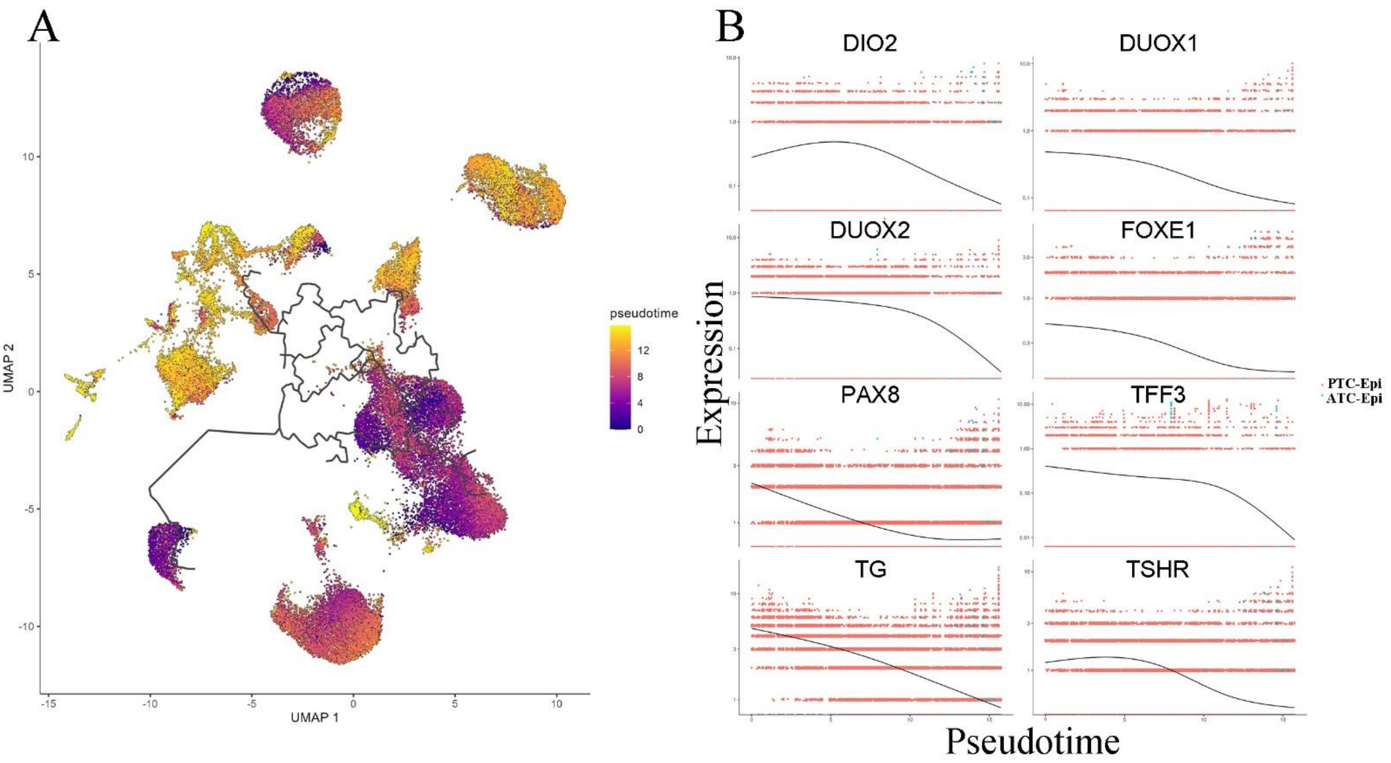

Supplemental Figure 5. Feature plots of markers genes for each subcluster of CAFs(A) and ECs(B).

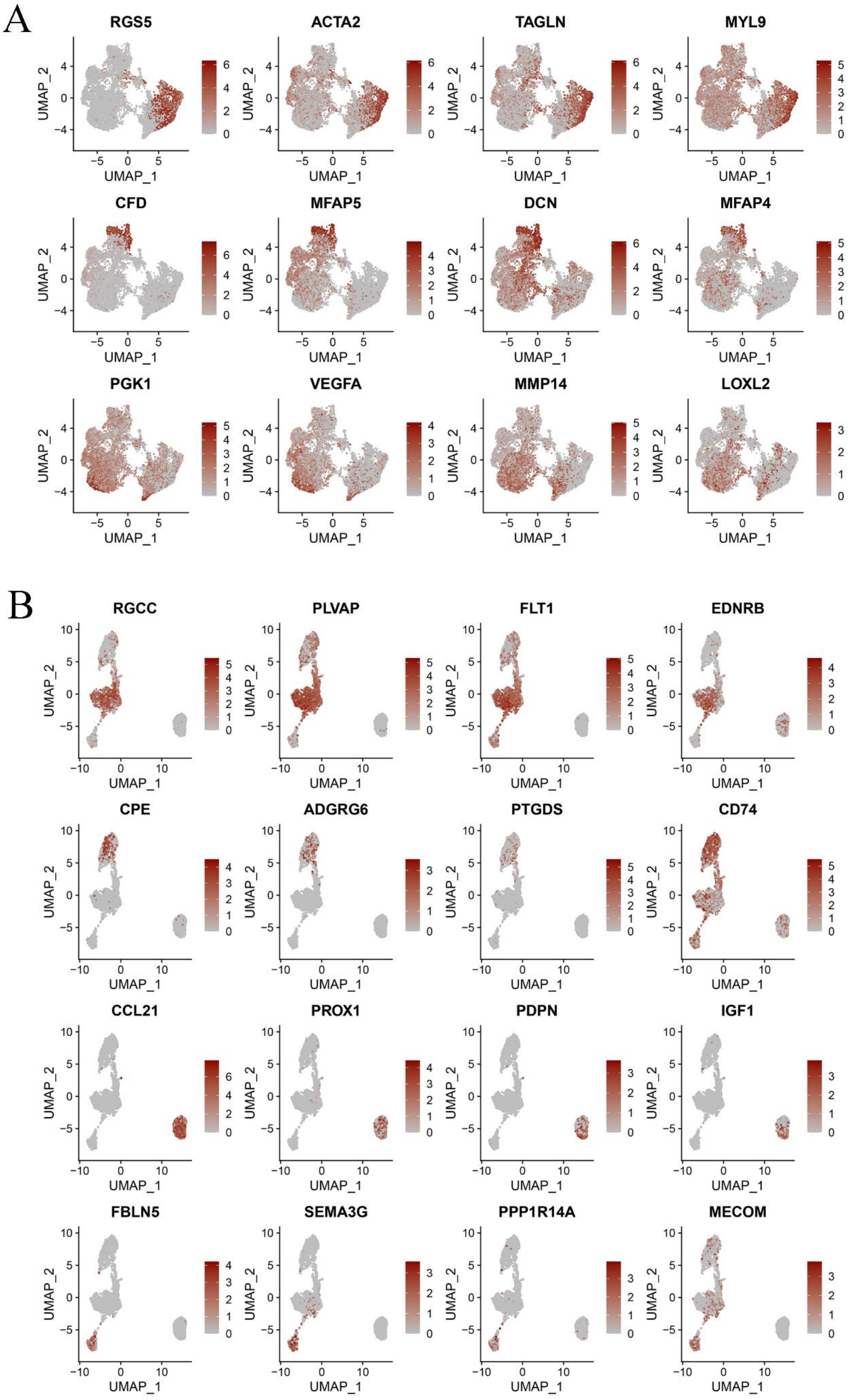

Supplemental Figure 6. Epithelial-stromal communication HT.

A. UMAP plot of cells from 4 HT patients

B. Specific marker genes for each major cell linages.

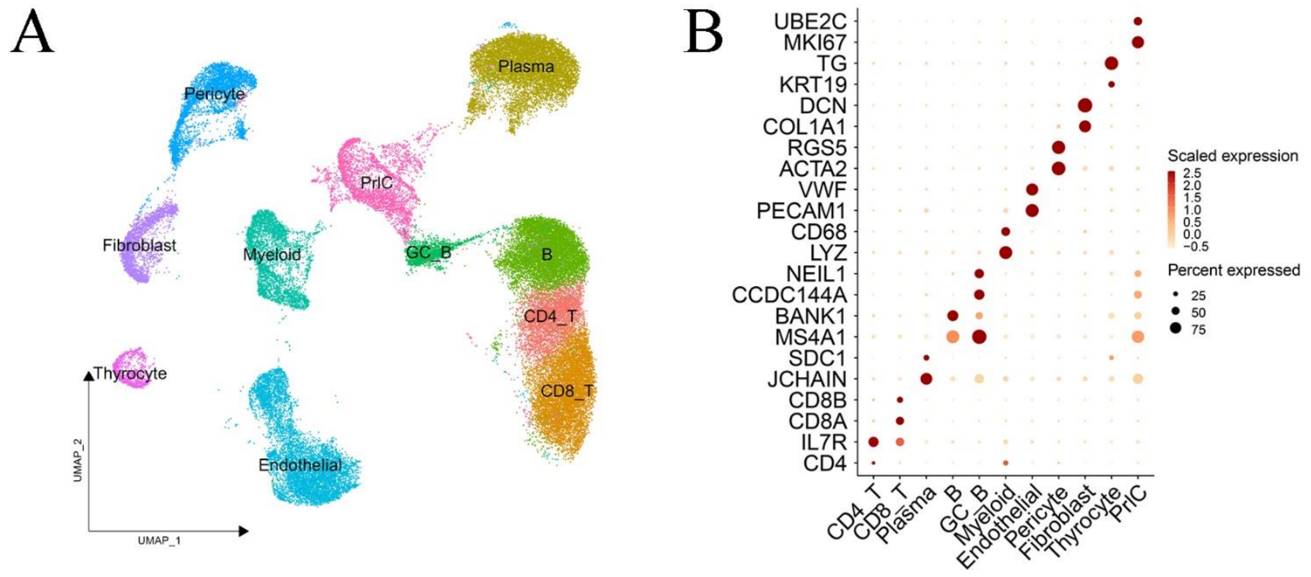

Supplemental Figure 7.   Different pattern of T/NK cells between ATC and PTC.

A. Heatmap of the top biomarkers for each subcluster of T/NK cells.

B. Bar plots demonstrated the percentage of twelve T/NK cell subclusters in different tissues.

C. Boxplots compared the percentage of each T/NK cell subcluster between ATC and PTC primary tumors. Wilcoxon test: *: $p < 0.05$; **: $p < 0.01$, ***: $p < 0.001$; ns: $p > 0.05$

D. Potential evolutionary trajectory of CD8 T cells colored by pseudotime (left) and cell identities (right).

See the next page.

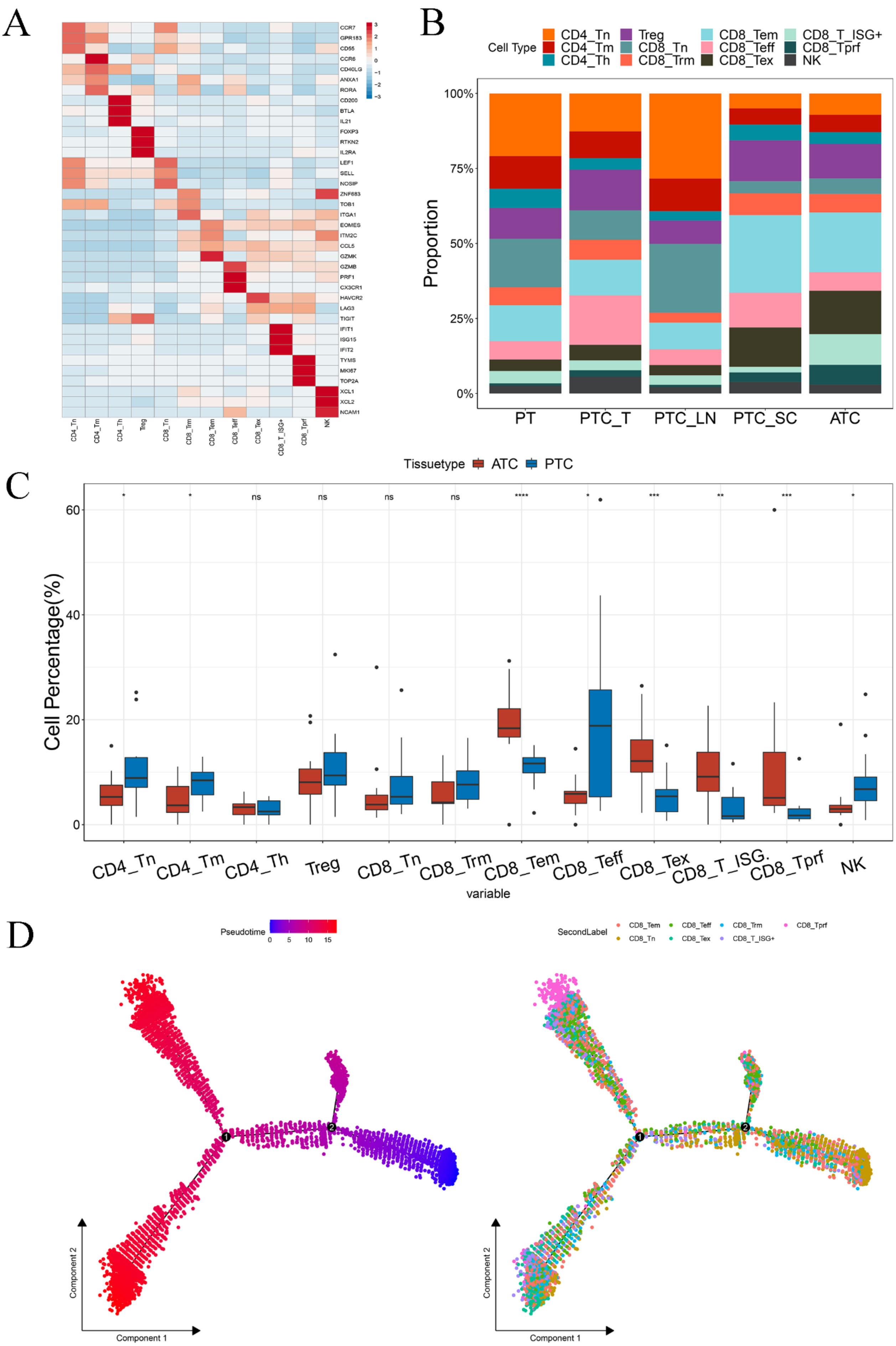
A
CCR7
GPR183
CD55
CCR6
CD40LG
ANXA1
RORA
CD200
BTLA
IL21
FOXP3
RTKN2
IL2RA
LEF1
SELL
NOSIP
ZNF683
TOB1
ITGA1
EOMES
ITM2C
CCL5
GZMK
GZMB
PRF1
CX3CR1
HAVCR2
LAG3
TIGIT
IFIT1
ISG15
IFIT2
TYMS
MKI67
TOP2A
XCL1
XCL2
NCAM1
3
2
1
0
−1
−2
−3
CD4_Tn
CD4_Tm
CD4_Th
Treg
CD8_Tn
CD8_Trm
CD8_Tem
CD8_Teff
CD8_Tex
CD8_T_ISG+
CD8_Tprf
NK
B
Cell Type
CD4_Tn
CD4_Tm
CD4_Th
Treg
CD8_Tn
CD8_Trm
CD8_Tem
CD8_Teff
CD8_Tex
CD8_T_ISG+
CD8_Tprf
NK
Proportion
100%
75%
50%
25%
0%
PT
PTC_T
PTC_LN
PTC_SC
ATC
C
Tissuetype
ATC
PTC
Cell Percentage(%)
60
40
20
0
*
*
ns
ns
ns
ns
****
*
***
**
***
*
CD4_Tn
CD4_Tm
CD4_Th
Treg
CD8_Tn
CD8_Trm
CD8_Tem
CD8_Teff
CD8_Tex
CD8_T_ISG.
CD8_Tprf
NK
variable
D
Pseudotime
0
5
10
15
SecondLabel
CD8_Tem
CD8_Teff
CD8_Trm
CD8_Tprf
CD8_Tn
CD8_Tex
CD8_T_ISG+
Component 2
Component 1
Component 2
Component 1

Supplemental Figure 8. Refined classification of myeloid cells.

A. UMAP plot of six myeloid cell subclusters.

B. Bar plots demonstrated the percentage of myeloid cell subclusters in different tissues.

C. Feature plots showed maker genes for myeloid cell subclusters

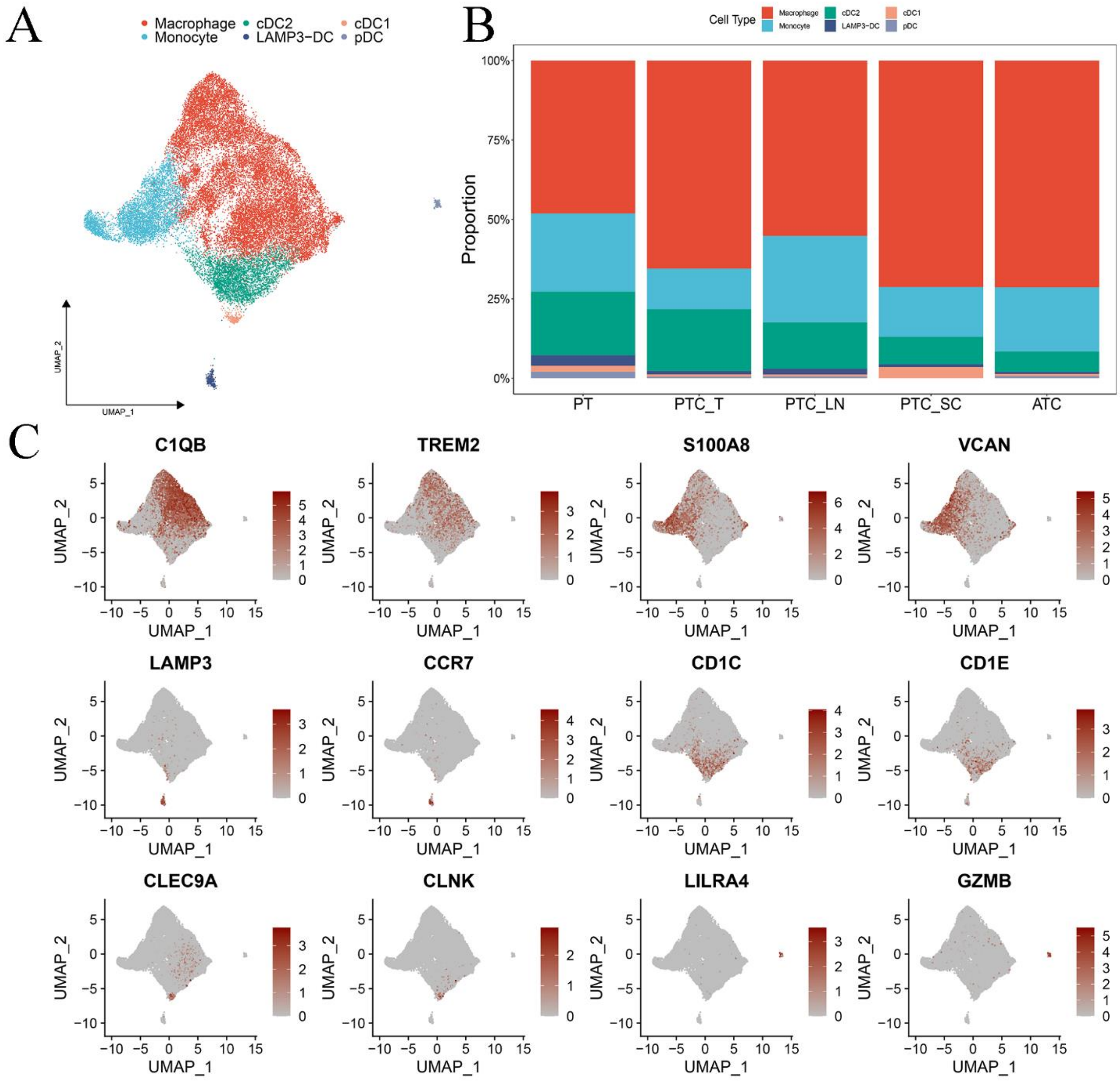

Supplemental Figure 9. Immune inhibitory molecules were upregulated in LAMP3-DC.

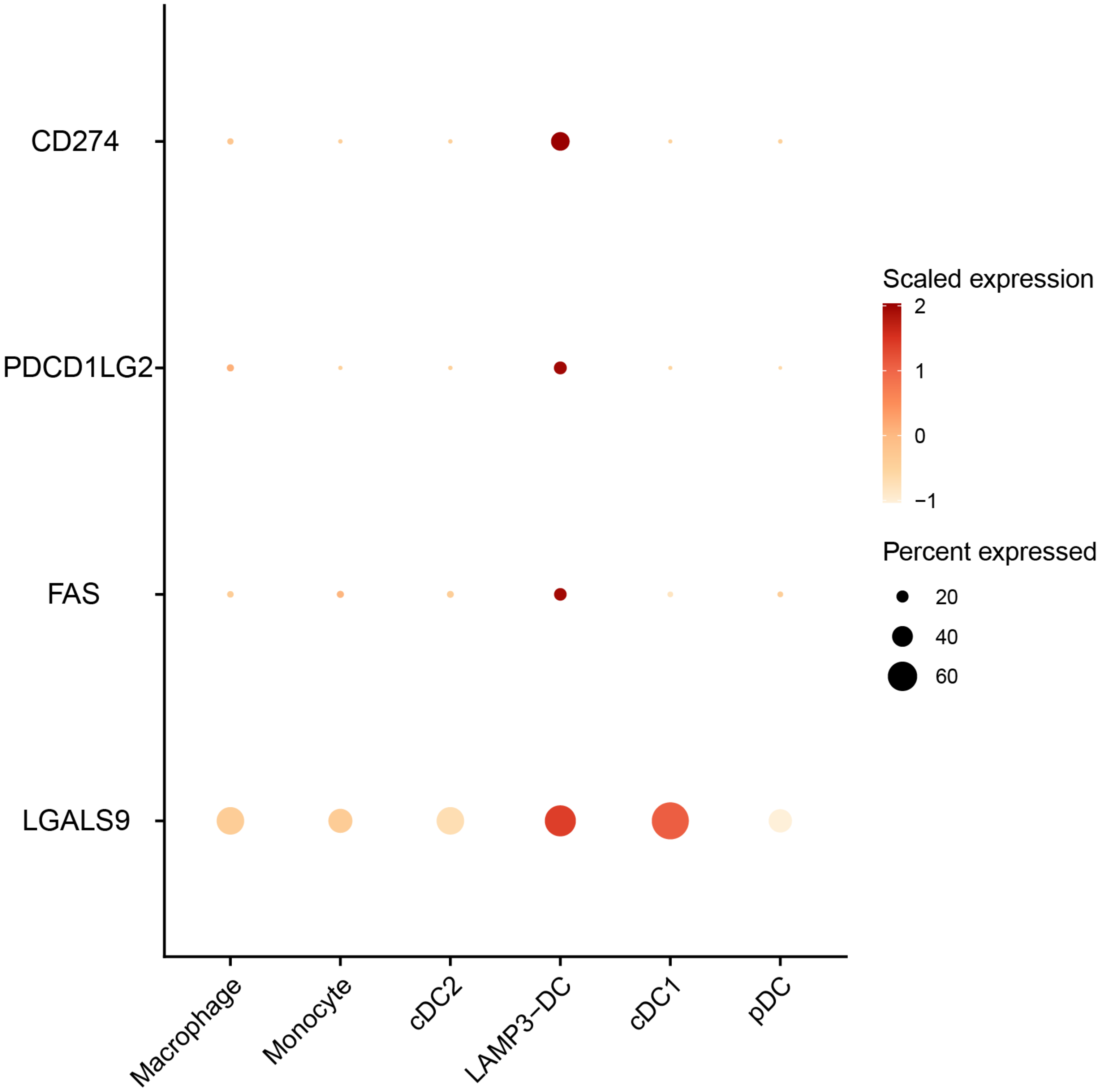

Supplemental Figure 10. The landscape of B lymphocytes in ATC and PTC

A. UMAP plot of four subclusters of B lymphocytes.

B. Feature plots showed maker genes for B cell subclusters. Bn: Naïve B Cells; Bmem: Memory B Cells; Plasma: Plasma: Plasma Cells; GC_B: Germinal Centre B Cells

C. Bar plots showed the percentage of B cells subclusters in different tissues

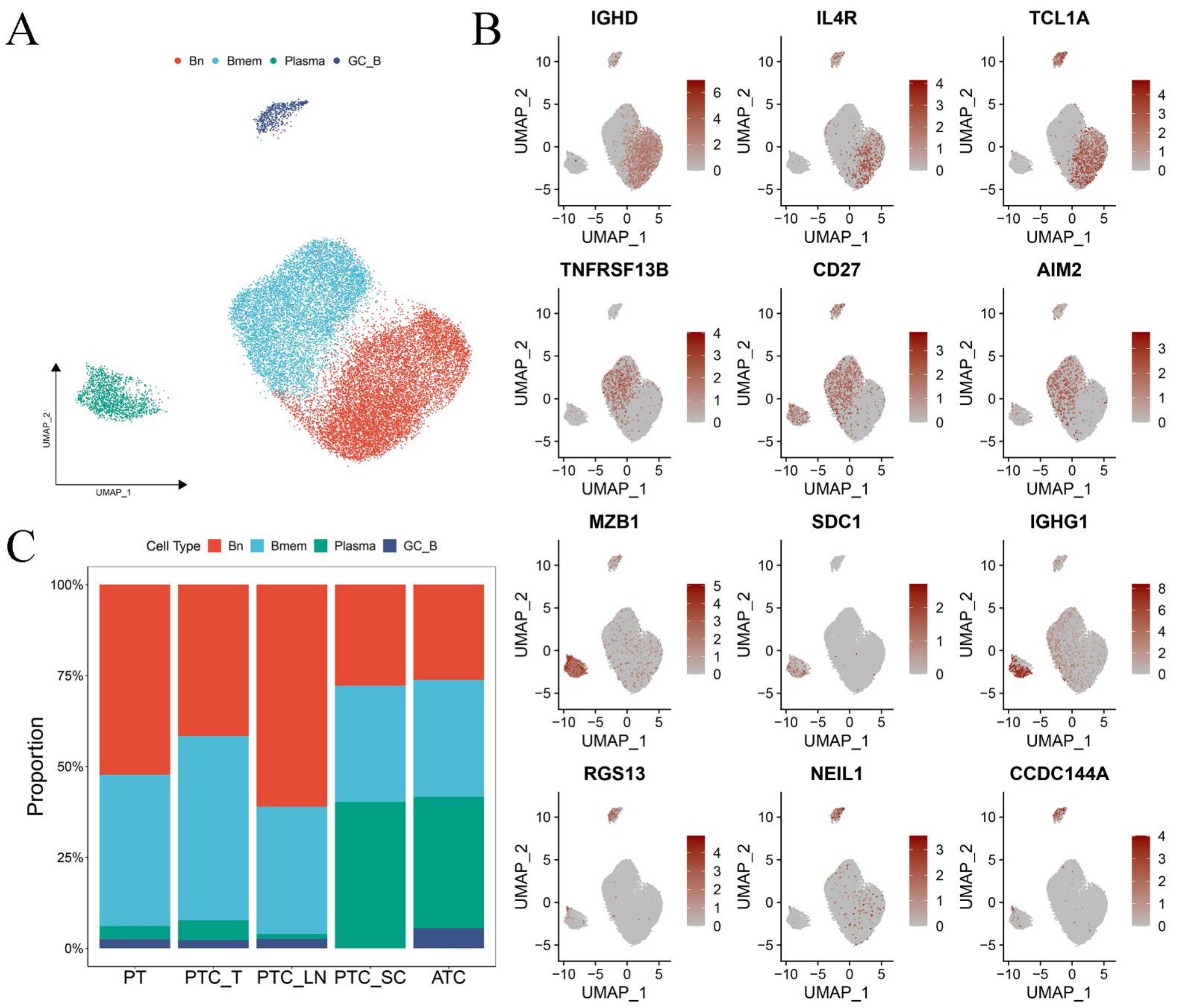

Supplemental Figure 11. ***CXCL13*** **expression of ATC, PDTC and PTC in the microarray datasets.** Student's t-test: *: $p < 0.05$; **: $p < 0.01$, ***: $p < 0.001$; ns: $p > 0.05$

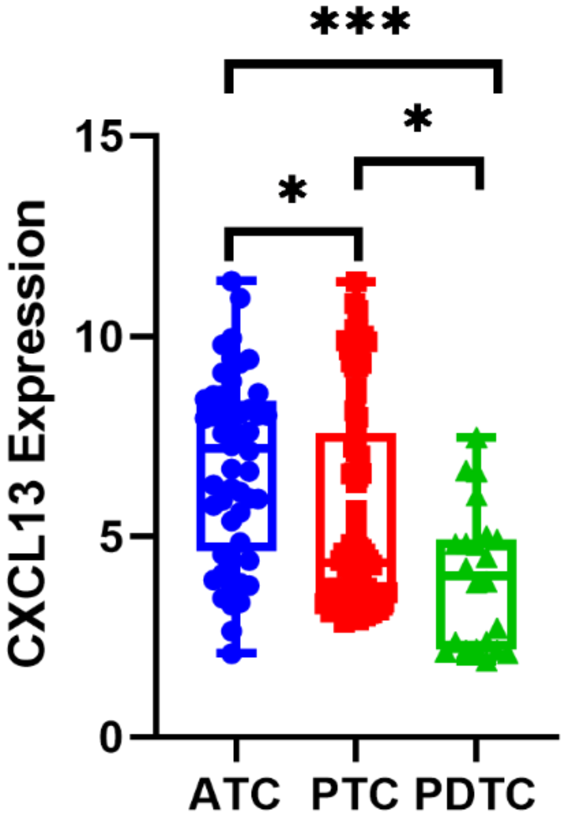

Supplemental Figure 12. Cell-cell interaction analysis revealed potential key axis for TLS formation in HT.

A. Cell–cell interactions between GC B cells and other cell lineages in HT. A key CXCL12-CXCR4 pair was highlighted with the red box.

B. Cell–cell interactions between immune cells and stromal cells in HT. A key CXCL12-CXCR4 pair was highlighted with the red box.

C. Dotplot of CXCL12 expression in stromal cells from HT samples and HT-free samples from our own scRNA-seq dataset. HT-No: Samples without Hashimoto's thyroiditis; HT-Yes: Samples diagnosed with Hashimoto's thyroiditis

D. Bar plots showed the percentage of B cells subclusters in the integrated scRNA-seq dataset. B cells took a larger percentage in the HT-positive samples than in HT-free samples.

See the next page.

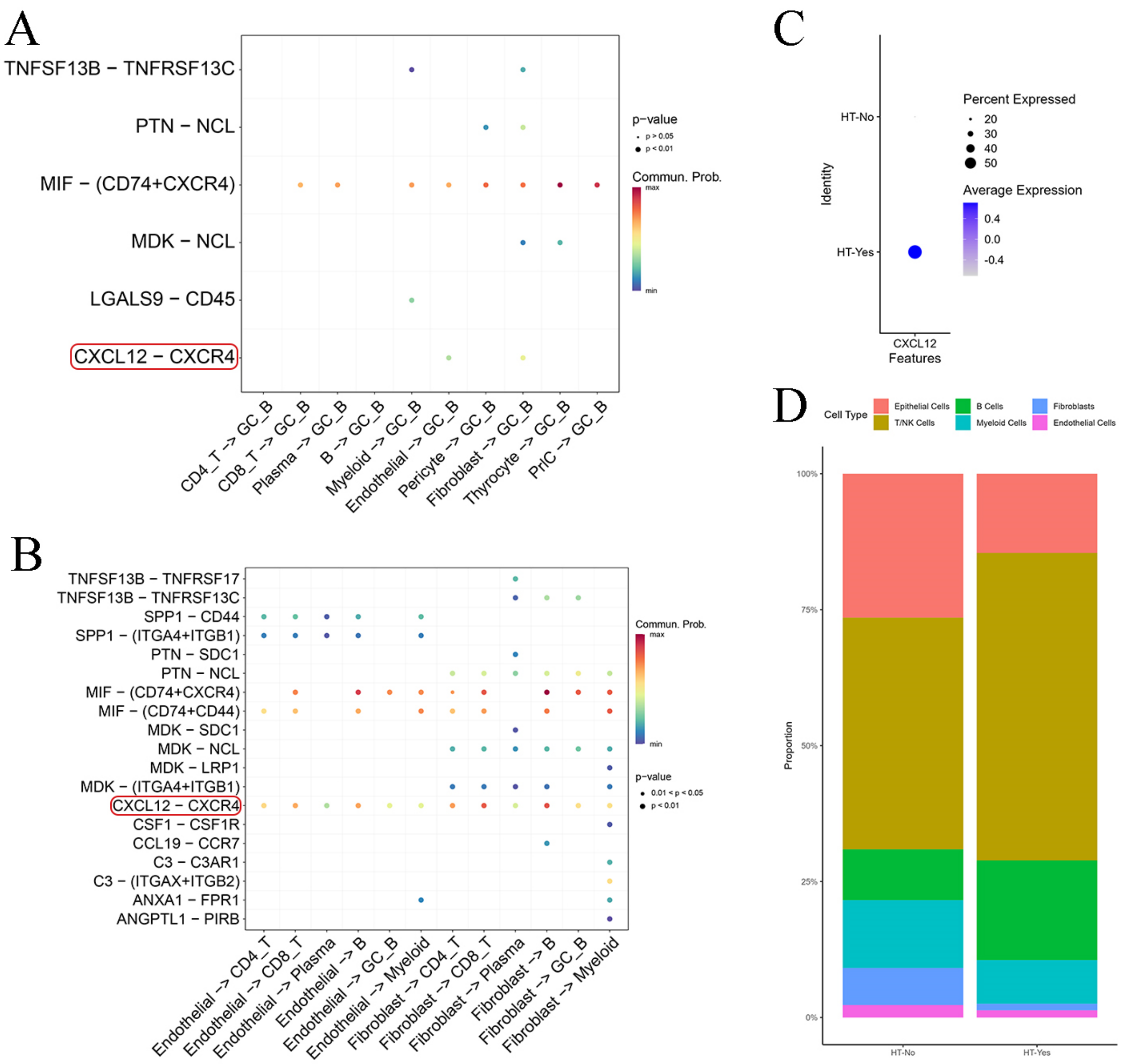
A
TNFSF13B – TNFRSF13C
PTN – NCL
MIF – (CD74+CXCR4)
MDK – NCL
LGALS9 – CD45
CXCL12 – CXCR4
p–value
p > 0.05
p < 0.01
Commun. Prob.
max
min
CD4_T -> GC_B
CD8_T -> GC_B
Plasma -> GC_B
B -> GC_B
Myeloid -> GC_B
Endothelial -> GC_B
Pericyte -> GC_B
Fibroblast -> GC_B
Thyrocyte -> GC_B
PrIC -> GC_B
B
TNFSF13B – TNFRSF17
TNFSF13B – TNFRSF13C
SPP1 – CD44
SPP1 – (ITGA4+ITGB1)
PTN – SDC1
PTN – NCL
MIF – (CD74+CXCR4)
MIF – (CD74+CD44)
MDK – SDC1
MDK – NCL
MDK – LRP1
MDK – (ITGA4+ITGB1)
CXCL12 – CXCR4
CSF1 – CSF1R
CCL19 – CCR7
C3 – C3AR1
C3 – (ITGAX+ITGB2)
ANXA1 – FPR1
ANGPTL1 – PIRB
Commun. Prob.
max
min
p–value
0.01 < p < 0.05
p < 0.01
Endothelial -> CD4_T
Endothelial -> CD8_T
Endothelial -> Plasma
Endothelial -> B
Endothelial -> GC_B
Endothelial -> Myeloid
Fibroblast -> CD4_T
Fibroblast -> CD8_T
Fibroblast -> Plasma
Fibroblast -> B
Fibroblast -> GC_B
Fibroblast -> Myeloid
C
HT-No
HT-Yes
Identity
CXCL12
Features
Percent Expressed
20
30
40
50
Average Expression
0.4
0.0
-0.4
D
Cell Type
Epithelial Cells
T/NK Cells
B Cells
Myeloid Cells
Fibroblasts
Endothelial Cells
Proportion
100%
75%
50%
25%
0%
HT-No
HT-Yes

Supplemental Figure 13. Construction of BPC, a murine thyroid cancer cell line

A. Schematic diagram of the development of BPC cell line.

B. Ultrasound image of a BPC mouse's thyroid. The thyroid tumor was circled by the green line.

C. Left: HE image of the primary thyroid tumor from a BPC transgenic mouse (15X); Right: Spotty TTF-1 (A marker of thyroid-specific differentiation) expression in the primary thyroid tumor from a BPC transgenic mouse (15X).

D. Left: HE image of a BPC xenograft (15X); Right: Spotty TTF-1 expression in the BPC xenograft (15X).

See the next page.

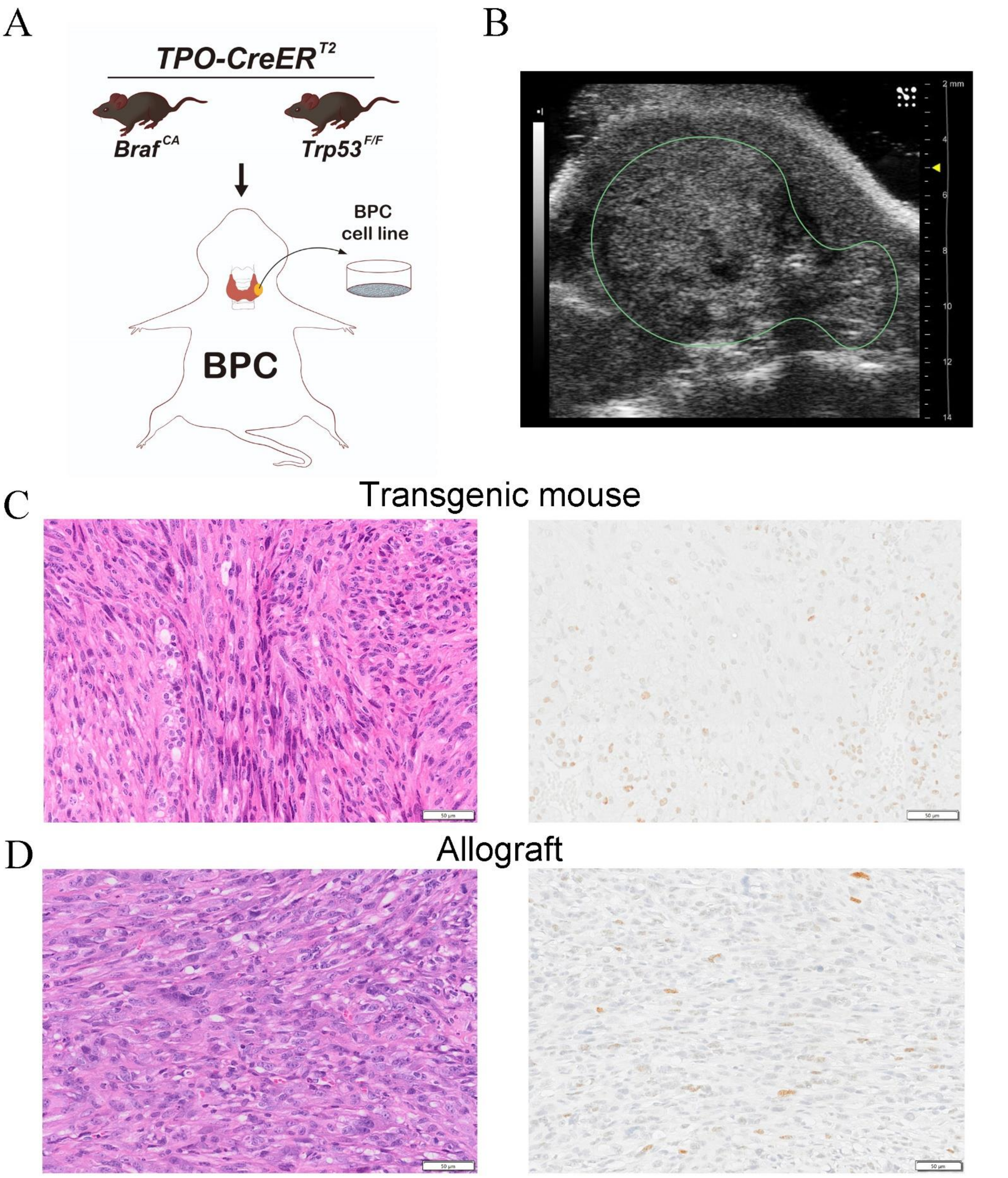
A
TPO-CreERT2
BrafCA
Trp53F/F
BPC cell line
BPC
B
2 mm
4
6
8
10
12
14
C
Transgenic mouse
50 µm
D
Allograft
50 µm

Supplemental Figure 14. Famitinib plus aPD-1 might potentiate anti-tumor action and TLS development in MC38 mouse xenografts.

A. Tumor images of the control group, anti-PD-1 (aPD-1) group and Famitinib plus aPD-1 (aPD-1+ Fami) group of MC38 xenografts.

B. Representative flowmetry images of each group of xenografts and the bar plot showed an increase of infiltrated CD19+ B cells in the aPD-1+Fami group

C. Growth curve and weight of control group, anti-PD-1 (aPD-1) group and Famitinib plus aPD-1 (aPD-1+ Fami) group of MC38 xenografts.

D. RT-PCR results indicated that some of the biomarkers related to TLS development were upregulated in the aPD-1+Fami group (*Vcam1* and *Cxcl13*), while *Icam1, Ltb*, and *Ltbr* showed no upregulation.

E. WB images and histograms suggested upregulation of Cxcl13 and MECA-79 in the aPD-1+Fami group. One-way ANOVA or Kruskal-Wallis test: *: $p < 0.05$; **: $p < 0.01$, ***: $p < 0.001$; ns: $p > 0.05$

See the next page.

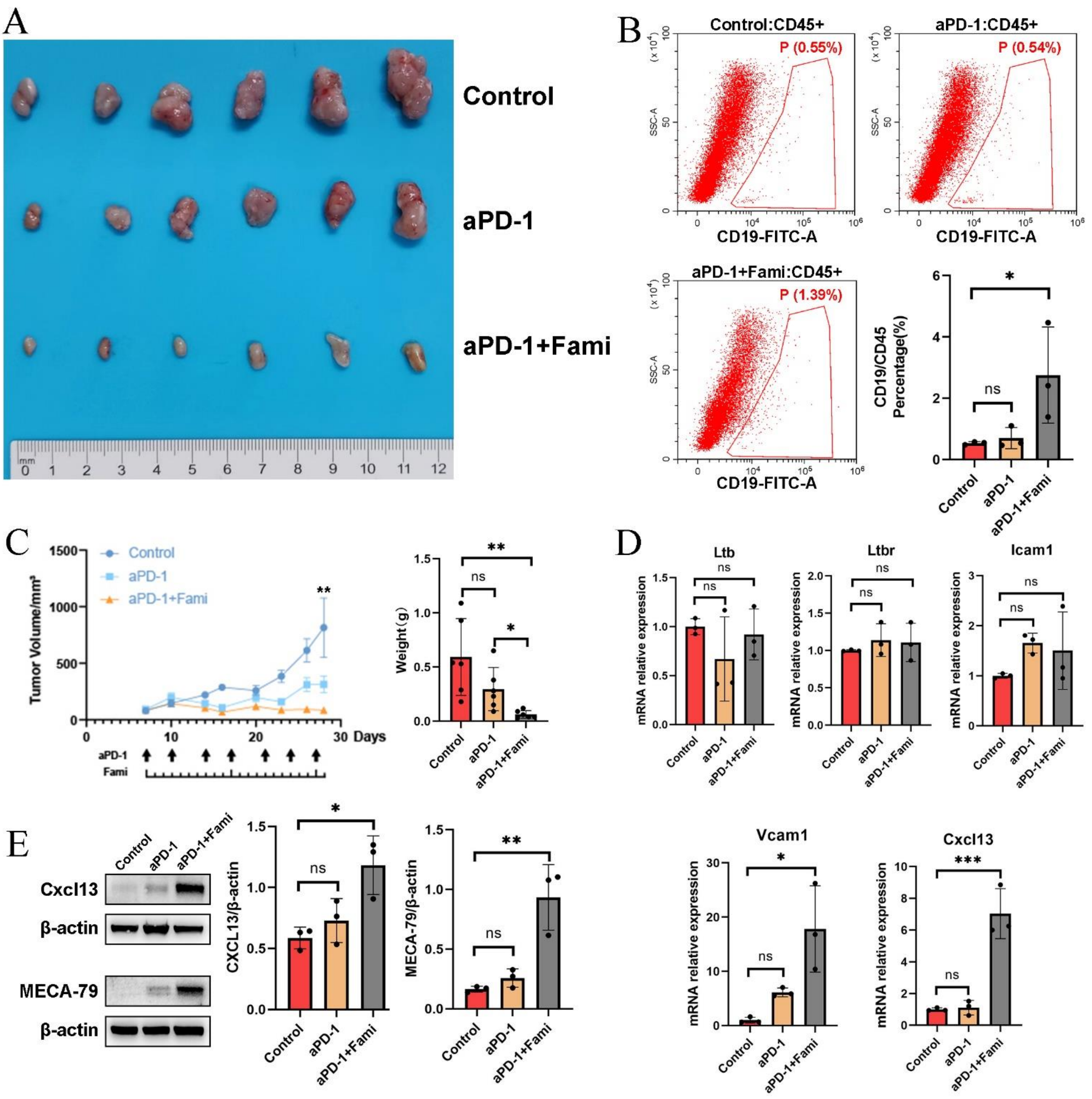
A
Control
aPD-1
aPD-1+Fami
B
Control:CD45+
P (0.55%)
aPD-1:CD45+
P (0.54%)
aPD-1+Fami:CD45+
P (1.39%)
SSC-A
CD19-FITC-A
CD19/CD45 Percentage(%)
ns
*
Control
aPD-1
aPD-1+Fami
C
Control
aPD-1
aPD-1+Fami
Tumor Volume/mm³
Days
**
aPD-1
Fami
Weight(g)
ns
*
**
D
Ltb
Ltbr
Icam1
Vcam1
Cxcl13
mRNA relative expression
ns
*
***
E
Cxcl13
β-actin
MECA-79
β-actin
CXCL13/β-actin
MECA-79/β-actin
ns
*
**

Supplemental Figure 15. Feature plots showed that key molecular inducers generally expressed in cells from Famtinib+Camrelizumab-treated A37 patient.

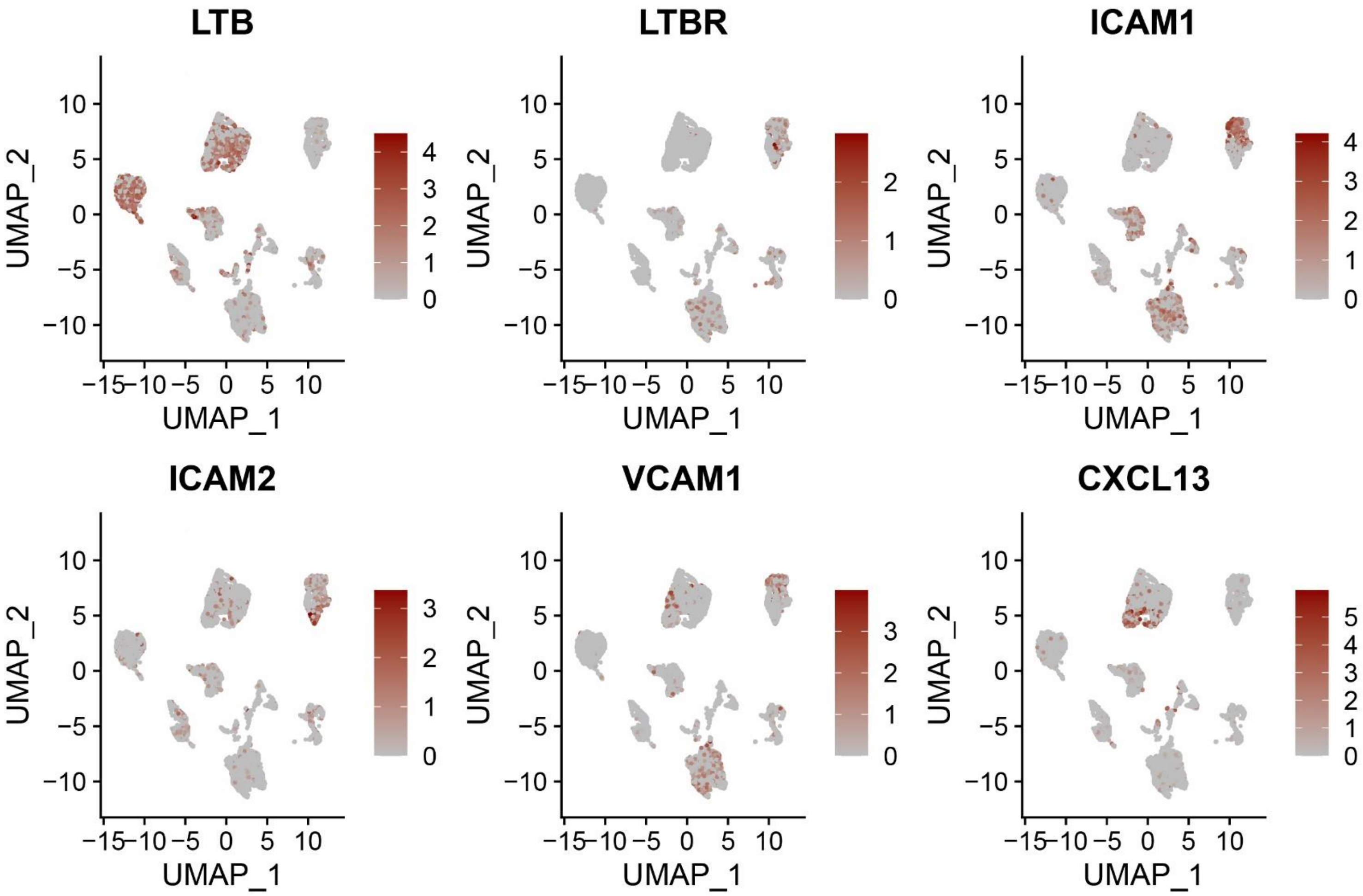

Supplement: Supplemental data [file jciinsight-9-173712-s021.pdf]
