# Supplementary material for: A distinct tumor microenvironment makes anaplastic thyroid cancer more lethal but immunotherapy sensitive than papillary thyroid cancer
Source: JCI Insight. 2024 Mar 13;9(8):e173712. doi: 10.1172/jci.insight.173712 (PMC11141884; doi:10.1172/jci.insight.173712)
Supplement: Unedited blot and gel images [file jciinsight-9-173712-s022.pptx]

## Slide 1
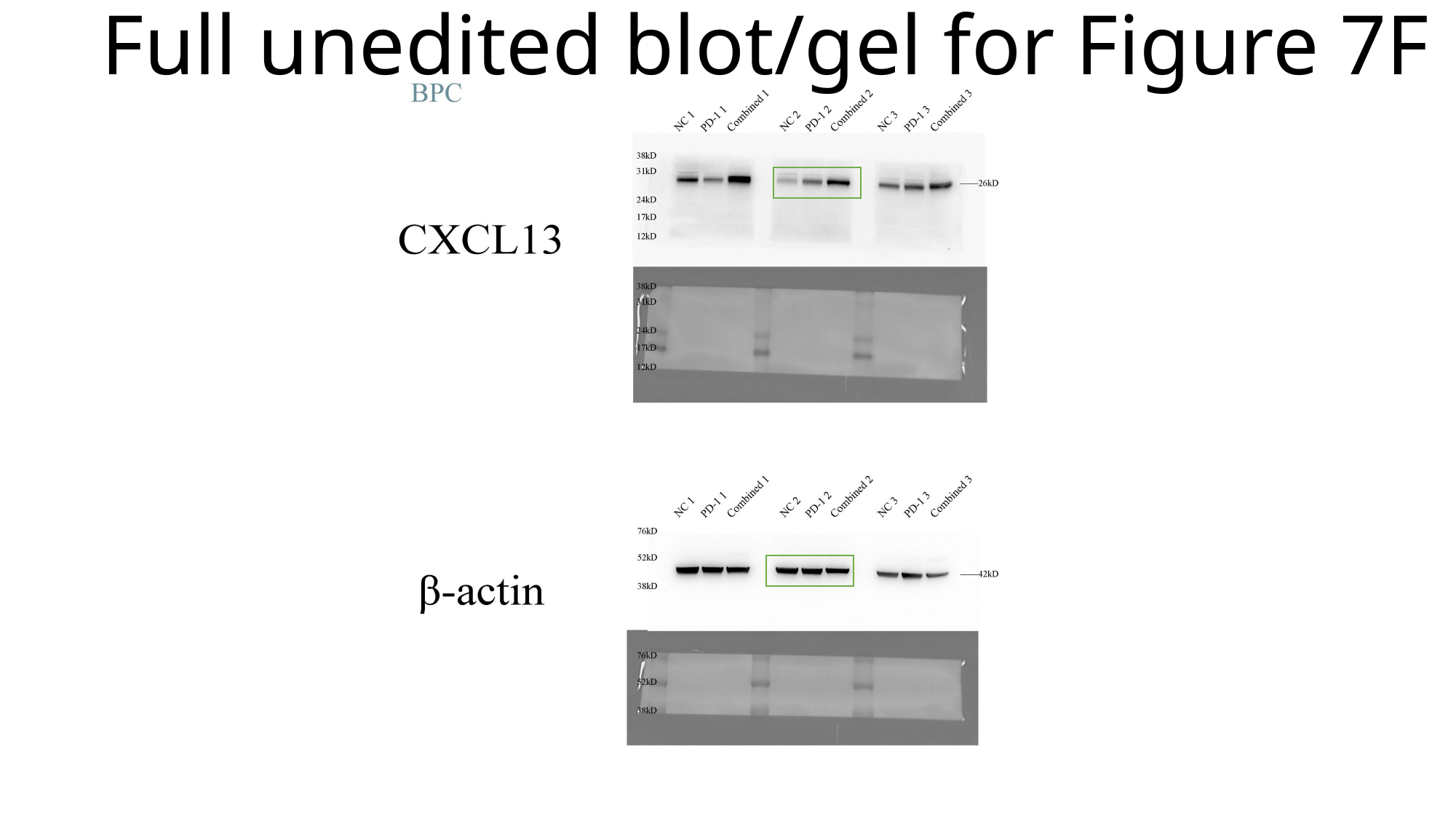

Full unedited blot/gel for Figure 7F

## Slide 2
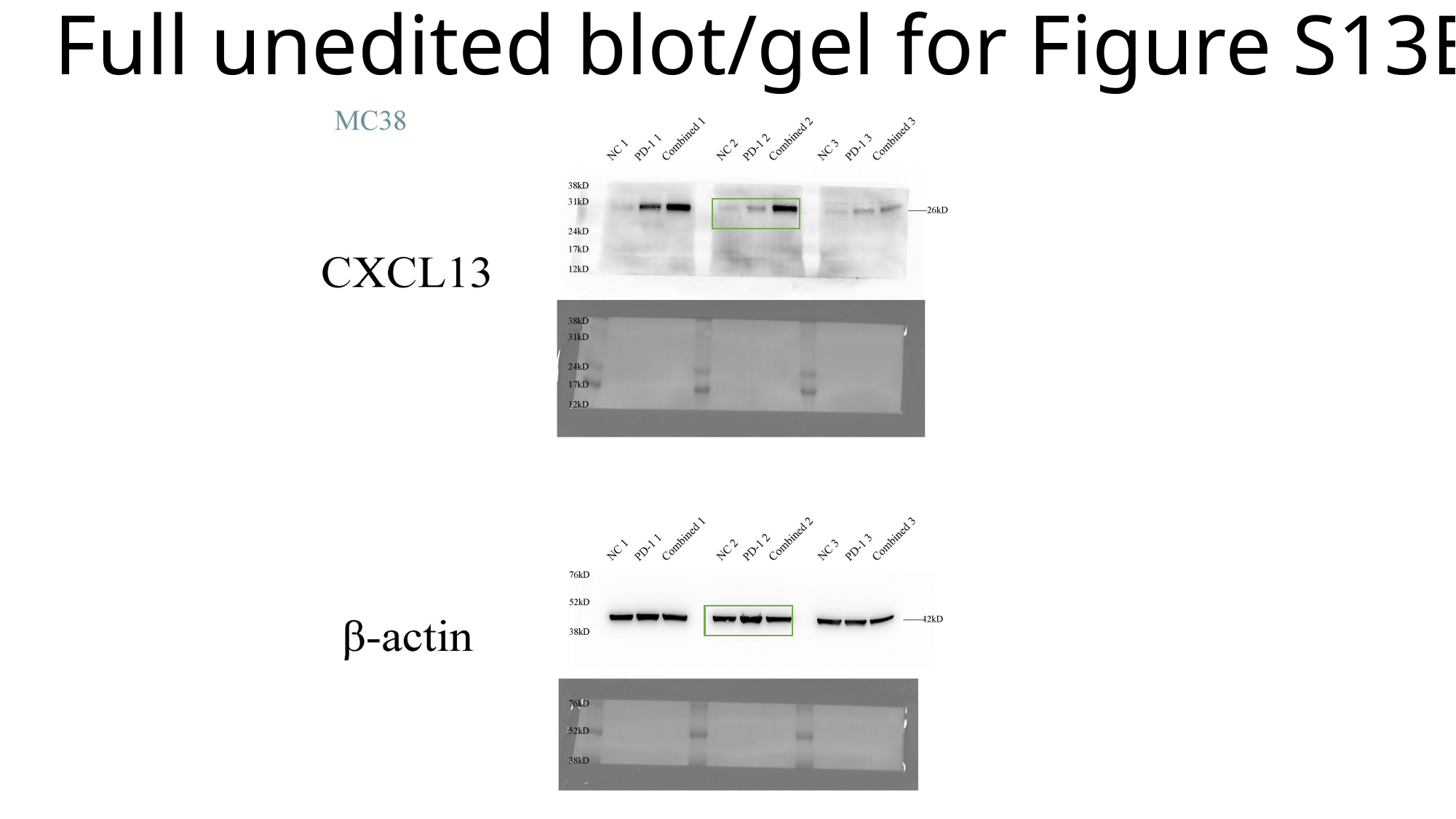

Full unedited blot/gel for Figure S13E

## Slide 3
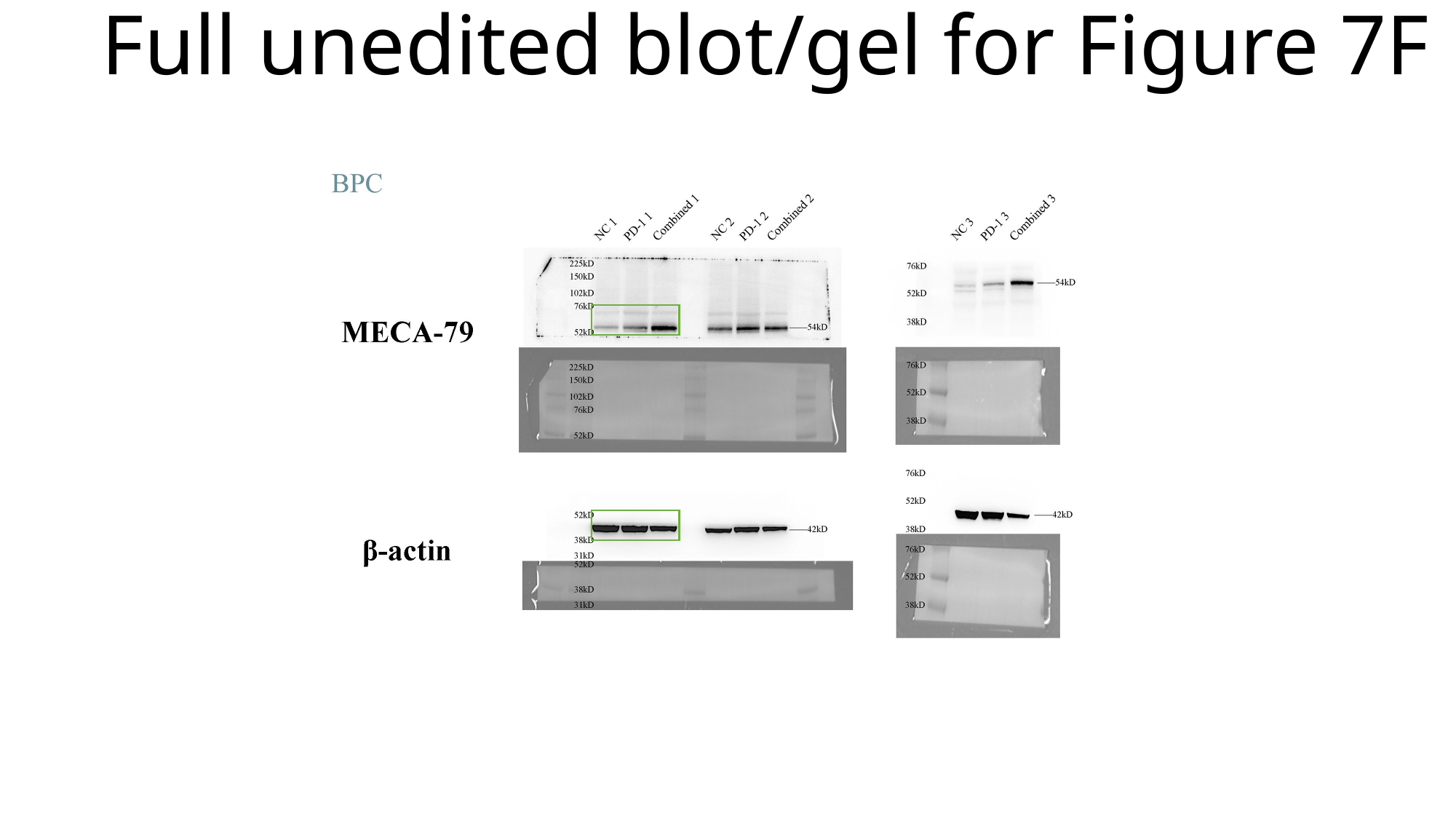

Full unedited blot/gel for Figure 7F

## Slide 4
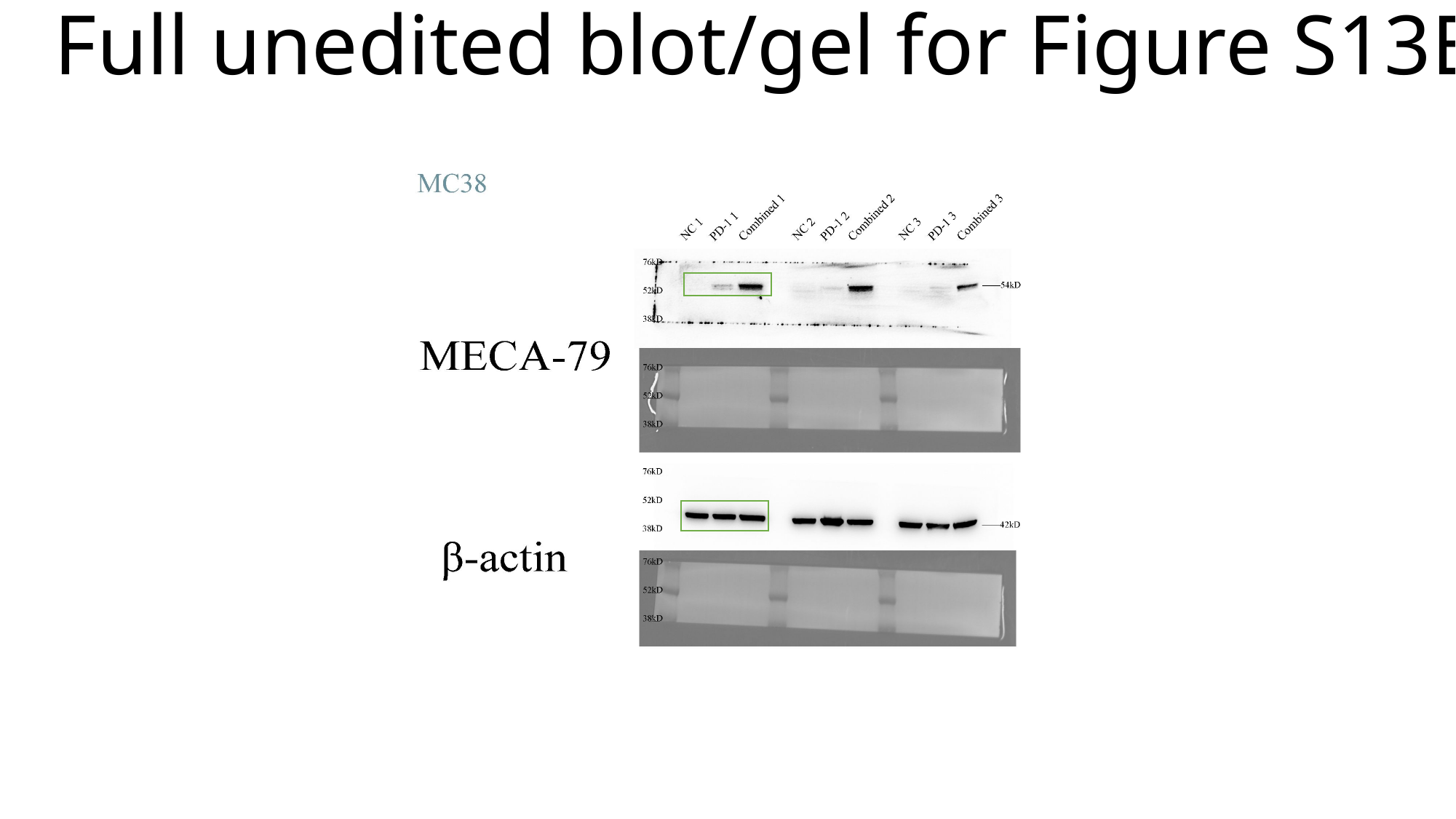

Full unedited blot/gel for Figure S13E
